# Transcriptomic studies and assessment of *Yersinia pestis* reference genes in various conditions

**DOI:** 10.1038/s41598-019-39072-x

**Published:** 2019-02-21

**Authors:** Lionel Koch, Thomas Poyot, Marine Schnetterle, Sophie Guillier, Estelle Soulé, Flora Nolent, Olivier Gorgé, Fabienne Neulat-Ripoll, Eric Valade, Florent Sebbane, Fabrice Biot

**Affiliations:** 1 Institut de Recherche Biomédicale des Armées (IRBA), Brétigny-sur-Orge, France; 2 Ecole du Val de Grace (EVDG), Paris, France; 3 Aix Marseille University, INSERM, SSA, IRBA, MCT, Marseille, France; 4 Inserm, University of Lille, CNRS, CHU Lille, Institut Pasteur de Lille, U1019-UMR8204-CIIL-Center for Infection and Immunity of Lille, Lille, France

## Abstract

Reverse transcription quantitative real-time polymerase chain reaction (RT-qPCR) is a very sensitive widespread technique considered as the gold standard to explore transcriptional variations. While a particular methodology has to be followed to provide accurate results many published studies are likely to misinterpret results due to lack of minimal quality requirements. *Yersinia pestis* is a highly pathogenic bacterium responsible for plague. It has been used to propose a ready-to-use and complete approach to mitigate the risk of technical biases in transcriptomic studies. The selection of suitable reference genes (RGs) among 29 candidates was performed using four different methods (GeNorm, NormFinder, BestKeeper and the Delta-Ct method). An overall comprehensive ranking revealed that 12 following candidate RGs are suitable for accurate normalization: *gmk*, *proC*, *fabD*, *rpoD*, *nadB*, *rho*, *thrA*, *ribD*, *mutL*, *rpoB*, *adk* and *tmk*. Some frequently used genes like *16S RNA* had even been found as unsuitable to study *Y*. *pestis*. This methodology allowed us to demonstrate, under different temperatures and states of growth, significant transcriptional changes of six efflux pumps genes involved in physiological aspects as antimicrobial resistance or virulence. Previous transcriptomic studies done under comparable conditions had not been able to highlight these transcriptional modifications. These results highlight the importance of validating RGs prior to the normalization of transcriptional expression levels of targeted genes. This accurate methodology can be extended to any gene of interest in *Y*. *pestis*. More generally, the same workflow can be applied to identify and validate appropriate RGs in other bacteria to study transcriptional variations.

## Introduction

Plague is a fatal disease caused by the Gram-negative bacterium *Yersinia pestis*^1^ which has been responsible for approximately 200 million deaths in the past and is still a global public health issue. Between 2010 and 2015, the World Health Organization (WHO) reported more than 3200 cases with more than 500 deaths^2^. In 2017, at least 64 patients succumbed during an outbreak in Madagascar^3^. Plague mostly circulates in rodent populations through the bites of infected fleas. Bubonic plague is the usual presentation in humans after fleabites while primary septicemia without detectable bubos is possible. Pneumonic plague may occur after bubonic and septicemic forms. At this stage, bacteria are transmissible by air, causing deadly primary pneumonic presentations^1^. Thus, life cycle of *Y*. *pestis* between the mammalian hosts through the flea vectors implies that the bacterium senses the oscillations of its environmental temperature. Therefore, it is not surprising that temperature controls several biological processes playing a key role in the colonization of both the mammalian host and the flea vector^4^. In fact, previous comparative transcriptomic and proteomic studies have shown that many genes are differentially expressed when temperature increases from 21/28 to 37 °C and inversely^5^.

Reverse transcription quantitative real-time polymerase chain reaction (RT-qPCR) is a widespread^6^ gold standard^7^ technique to explore transcriptional variations. It is often the easiest and the most cost effective solution to measure the genes’ expression^8^ and to understand complex regulatory networks. Moreover, the expansion of the RNA sequencing (RNA-seq) did not replace the RT-qPCR because these two techniques work complementarily^7^. RNA-seq identifies the most relevant genes and RT-qPCR validates its results^9^, especially in the field of environmental and host adaptation^10,11^ and antimicrobial response^12^.

This specific and sensitive approach requires a very particular methodology^13^ to provide with accurate and reliable results^14^. Unfortunately, Bustin *et al*.^15^ demonstrated that the majority of published studies did not fulfill all requirements even in the highest impact factor revues. Indeed, the whole workflow must be considered^16^ and drastic quality controls have to be performed at each step^17^. With highly pathogenic agents as *Y*. *pestis*, the method of RNA extraction must be carefully chosen^18^ for biosafety concerns as well as to preserve the RNA quality, essential for further reactions^19^. The reverse transcription step transforming extracted RNA into cDNA is critical for the accuracy of the qPCR, which measures the relative quantity (RQ) of cDNA^20^. However, considering numerous technical specifications can minimize the variability^21^. The knowledge of the PCR efficiency and the use of controls are necessary to guarantee the quality and the reproducibility of the results^22^. A normalization strategy is essential to mitigate the technical variations for qPCR during the previous steps, and then enabling comparisons between different samples.

The use of reference genes (RGs) is the most commonly accepted method for normalizing^23^. The ratio of the mRNA concentrations of the genes of interest is reported to those of the RGs, which are considered as stably expressed. The stability of this expression is evaluated with different algorithms, which are elaborated in the method section. However, a suitable and universal set of RGs does not exist and every investigator should select and analyze candidate RGs for every experimental conditions^13^. Several sets of candidate genes have already been evaluated for different bacteria^24–26^ but to our best knowledge, RGs have not been investigated in *Y*. *pestis*. This highly regulated pathogen is an excellent model for studying the variation of its genes expression in response to environmental changes. Our experimental parameters have been chosen to maximize the transcription variability in the reputed stable genes, and hence to ensure our choice of RGs in a broad spectrum of physiological conditions.

**Figure 1:**
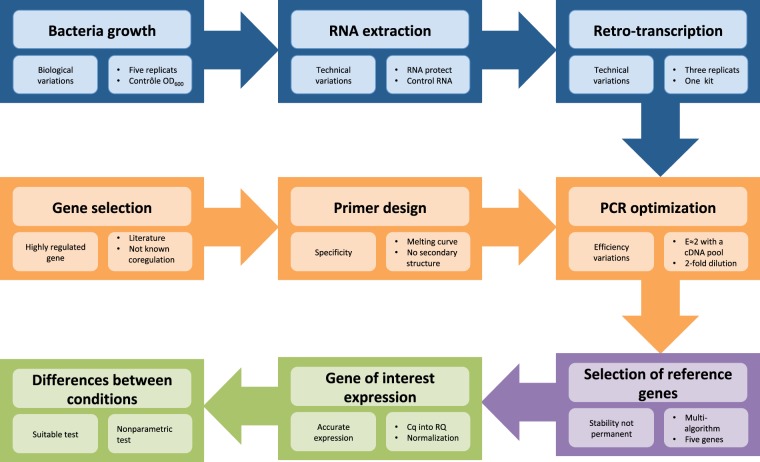
Workflow for validation of RGs and transcriptomic studies. Blue section presents the three steps to obtain suitable cDNA and control its quality. Orange section describes the method to choose pertinent candidates RGs and to obtain suitable PCR conditions from the primers design to the PCR parameters’ optimization (E = 2 corresponds to 100% efficiency and E = 1 to 0%). Purple section shows the selection of the appropriate number of stable enough RGs using multiple algorithms. Green section displays the normalization of the gene of interest’s expression and the use of the appropriate statistic tests to identify differences.

We identified the most appropriated combination of RGs, which could be used for any transcriptional study in *Y*. *pestis* including transcriptomic analysis using RNA-seq. We highlight that this methodology could also be a starting point for a similar study in other species. Furthermore we present, through a workflow strictly applying the MIQE (Minimum Information for Publication of Quantitative Real-Time PCR Experiments) recommendations^13^ (Fig. 1), a global approach reducing bias for transcriptomic studies in bacteria. These consensual guidelines describe how to perform and interpret qPCR experiments in order to ensure the reliability and the transparency of the results. Lastly, as a proof-of-concept, we used this set of genes to perform an accurate normalization of six Resistance-Nodulation-Division (RND) efflux pumps gene expression in *Y*. *pestis* in function of the temperature and the growth phase. These genes have been selected because they have been reported in other bacteria to be implicated in a wide range of physiological aspects as antimicrobial resistance, bacterial stress responses, fitness, colonization and virulence^27^.

## Results

### Expression level of the candidate RGs

**Figure 2:**
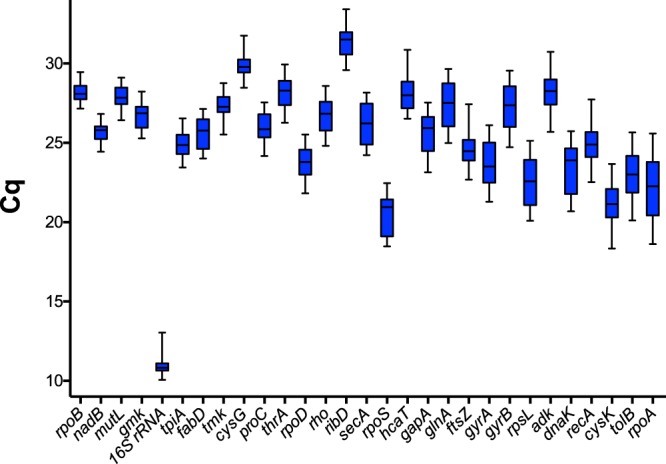
Expression level for the 29 candidate reference genes in all samples. Expression level was represented by the C_q_ values of each gene, ranked from the smallest to the largest range. Ebox indicated the 25th and 75th percentiles and the whiskers caps represented the maximum and minimum values. The line across the boxes indicated the median.

A total of 29 candidate RGs were selected (Table 1) on the basis of our laboratory experience and of an extended literature screening of genes designated as “housekeeping” gene in *Yersinia* genus^28,29^ and other genes commonly used as RGs in other bacteria species and present in *Y*. *pestis* genome^24–26,30^. In order to avoid co-regulation induced bias, we chose genes with various functions, distributed throughout the entire chromosome (Fig. S1) and not belonging to the same operon. The quantification cycle (C_q_) values from each of the 29 candidate RGs were generated by RT-qPCR using RNA extracted from exponential and stationary-phase of *Y*. *pestis* grown at 21 °C, 26 °C and 37 °C in Luria-Bertani (LB) broth. Results are represented in a box-and-whiskers plot (Fig. 2). Position and dispersion parameters are represented in Table S1. Analysis showed consequent differences between each selected candidates, indicating a differential expression depending on the experimental conditions and an internal variability. The C_q_ value medians ranged from 20.95 for *rpoS* to 31.52 for *ribD* except for *16S rRNA* for which the median was found at 10.83. A C_q_ value interquartile gap was observed from 0.42 [10.65; 11.07] for 16S rRNA to 3.31 [20.47; 23.78] for *rpoA*. A C_q_ value variation of 1 corresponds to the doubling of RNA quantity and only five genes (*16S rRNA*, *nadB*, *cysG*, *rpoD* and *tmk*) displayed an interquartile gap equal or less than 1. The range varied between 2.3 [27.16; 29.46] for *rpoB* and 6.97 [18.62; 25.59] for *rpoA*. The C_q_ value of only five genes (*rpoB*, *nadB*, *mutL*, *gmk* and *16S rRNA*) had a range 3, that corresponds to a 8-fold increased RNA quantity. Under all growing conditions, C_q_ value variations remain important even for genes with the lowest dispersion parameters.

**Table 1:** Primers for candidate reference genes and for *acrB* and the five putative RND efflux pumps genes of *Yersinia pestis*.

Gene symbole	Locus tag	Gene description	primer 5′-3′	
**Candidate reference gene**				
*16S rRNA*	YPOr01	16S ribosomal RNA	Fw	AGAGATGCTAAAGTGCCTTC
			Rv	CCAACATTTCACAACACGAG
*glnA*	YPO0024	glutamine synthetase	Fw	GCTGAACATGTTTTGACGATGCT
			Rv	TGCTCTTTCCCTTTGGTATCAGT
*gmk*	YPO0040	guanylate kinase	Fw	TTTTCATCTTGCCGCCATC
			Rv	GATAACTTCTTCGCTATCCTG
*tpiA*	YPO0085	triosephosphate isomerase	Fw	ATCCGTGACCATATCGCCAAG
			Rv	TTTCAGTGATGCACCGCCAAC
*cysG*	YPO0158	uroporphyrin III C-methyltransferase	Fw	GAAAAGTTGCTGACCCACGAC
			Rv	TGCACCGACCAACACCAC
*rpsL*	YPO0200	30S ribosomal protein S12	Fw	ACTAACGGTTTTGAAGTCAC
			Rv	TCTTTAACACGACCGCCAC
*rpoA*	YPO0234	DNA-directed RNA polymerase, alpha subunit	Fw	CTGAAAACGCCGAACCTG
			Rv	ATGCCTAAAGAAAGACCAC
*mutL*	YPO0371	DNA mismatch repair protein	Fw	GTTTCTTTACCATTACGCCAAC
			Rv	TGGCGATATCTCTTCATGCTG
*thrA*	YPO0459	bifunctional aspartokinase I/homoserine dehydrogenase I	Fw	TGTCCGGTTCACTTTCCTTC
			Rv	ATACCCCAACGCCTTAGCTT
*dnaK*	YPO0468	molecular chaperone DnaK	Fw	AGGCTGTCACTAACCCTCA
			Rv	CGCTGTGCTTCTTCGTCT
*ftsZ*	YPO0560	cell division protein FtsZ	Fw	GCCATCTCCAGTCCGTTG
			Rv	ATAGTGTTACCCACGGTCTC
*secA*	YPO0564	protein translocase subunit SecA	Fw	ATCAACCGCATGGAACCTG
			Rv	TTTCGCTAAACGCTCACGGAAC
*rpoD*	YPO0643	RNA polymerase sigma factor	Fw	AAGACGGTATCAATCAGGTTC
			Rv	ATTCGCCCGCTTCAACAC
*proC*	YPO0942	pyrroline-5-carboxylate reductase	Fw	TAAAGCCCCAGTTAATGGCCGATG
			Rv	ACCAACTTATCGCTAAAATCGACCTG
*tolB*	YPO1124	translocation protein TolB	Fw	TCTGATGTCACCCGCTTGGTC
			Rv	AATAACCAATGCTGATTTGCCAC
*gyrA*	YPO1216	DNA gyrase A	Fw	CAGTGGCAGAATATCCAAC
			Rv	TTGAACAGCACCGACAAC
*fabD*	YPO1598	malonyl-CoA transacylase	Fw	ATAATCCGGTACGTTGGACTG
			Rv	AGGCCGGTCAATACTTTACCTG
*tmk*	YPO1605	thymidylate kinase	Fw	CCTTTAGCGGAAAAACTGCGTGA
			Rv	GGCGGCATACAGCATTAATACCT
*gapA*	YPO2157	glyceraldehyde-3- phosphate dehydrogenase	Fw	GAAACTGCACGTAAGCAC
			Rv	TTAACGCCCATAACGAAC
*nadB*	YPO2710	L-aspartate oxidase	Fw	GTATTGCCGCTGTTTTCGAC
			Rv	TGGCAATAAATTCAACGGCTTC
*hcaT*	YPO2904	3-phenylprop- ionic transporter	Fw	CTTAATGGCTTCAACGAC
			Rv	CAGACAGTAAACGAACCAC
*cysK*	YPO2992	cysteine synthase A	Fw	CGGTCAAGAGATCAAACCTG
			Rv	AATGCCTTCTTCATCCATC
*adk*	YPO3118	Adenylate kinase	Fw	ATCACGTTAAATTCAACCCAC
			Rv	CTTACGGACAGTCGCTTC
*ribD*	YPO3183	bifunctional diaminohydroxyphosphoribosylaminopyrimidine deaminase/5-amino-6-5-phosphoribosylaminouracil reductase	Fw	GAAACGGAATATTAACTCTG
			Rv	CTAGCTCGCATAATCCAC
*recA*	YPO3307	recombinase A	Fw	TCCAGATCCTCTACGGTG
			Rv	CGCCAGCTTTCTCAATCAG
*rpoS*	YPO3355	RNA polymerase sigma factor RpoS	Fw	GCCGAGAAATTGGTTTGACAC
			Rv	AACAACGCCTCGATGCTCAG
*rpoB*	YPO3747	DNA-directed RNA polymerase, beta subunit	Fw	CCTTTAGCGGAAAAACTGCGTGA
			Rv	ACATTTTCTACTAATTGCACCCT
*rho*	YPO3867	rho termination factor	Fw	AAGAAGTAACCGAGATGCAAC
			Rv	TTTCAGCGACTTGAACGTGAC
*gyrB*	YPO4094	DNA gyrase B	Fw	GACTATCGCCCGCATGGAAC
			Rv	ACCTCTTTATTGCCCAAATCCTC
**Gene of interest**				
/	YPO0421	multidrug efflux protein	Fw	CAGTATTACATTGGTGCTC
			Rv	TATCCGTACCATAAGGCAG
/	YPO1001	integral membrane efflux protein	Fw	ATGACCGCTATTTCGTTTATCCTC
			Rv	ATGCCGCCAAATACCGTG
*mdtB*	YPO2848	multidrug efflux system subunit	Fw	ACAAGACAACGGGCTGATTC
			Rv	CTACTGCCGGATCTTTGAGG
*acrD*	YPO3043	aminoglycoside/multidrug efflux system	Fw	CGTCGATGCCTTTGGTTCACA
			Rv	CGCACTGACAATATCACTCGT
*acrB*	YPO3133	multidrug efflux protein	Fw	CGTACCCAGAAAGTACTGAACCA
			Rv	CCGGTGTTCTGACCTTGACCA
/	YPO3482	multidrug efflux protein	Fw	GTGATGTGGATACCCGCTCT
			Rv	CCGTAAATCCAGCGAGTTGT

For each temperature (21; 26 and 37 °C), RNAs have been extracted at exponential and stationary phases generating 6 groups. The intra-group variability was observed as drastically decreased with a C_q_ value interquartile gap between 0.16 (*glnA*; 21 °C; exponential; [25.71; 25.87]) and 1.42 (*cysK*; 37 °C; exponential; [19.10; 20.52]) and a range between 0.56 (*dnaK*; 21 °C; exponential; [21.26; 21.82]) and 4.01 (*gapA*; 26 °C; exponential; [23.14; 27.15]). The C_q_ value of five genes (*16S rRNA*, *rpoD*, *fabD*, *rho* and *gyrB*) had an interquartile gap equal or less than 0.5 in each sub-group (Fig. S2). Position and dispersion parameters in each sub-group are represented in Table S2.

### Evaluation of candidate RGs stability

The position and dispersion parameters provided an overview of the disparity in expression level and internal variation of genes expression. However, these parameters based on absolute data are not sufficient to evaluate the stability of the expression of each candidate RG. Hence, relative expression has to be taken into account by statistical analysis in order to assess gene stability^31^. Four methods (GeNorm^32^, BestKeeper^33^, NormFinder^34^, and the delta-Ct method^35^) have been used to rank the 29 candidate RGs. BestKeeper is the only one, which evaluates the stability of each gene independently of each other. Each algorithm provides a different ranking (Table 2). However, several genes were considered to be stable by different methods with overlapping ranks. Indeed, based on the results from GeNorm^32^ and considering that a M-value lower than 0.7 is the cut-off value to select a suitable reference gene, 15 genes were considered stable: *rpoD*/*proC*, *fabD*, *gmk*, *rho*, *thrA*, *adk*, *ribD*, *nadB*, *mutL*, *tmk*, *rpoB*, *tpiA*, *ftsZ* and *secA* (in descending order with regard to stability). With the exception of *secA*, all the above cited genes as well as *cysG*, *hcaT*, *recA*, *cysK* and *gapA* were also found to be suitable using BestKeeper algorithm and based on the authors recommendations (cut-off value of the standard deviation lower than 1)^33^. Although there is no cut-off value or recommended number of genes for the NormFinder^34^ algorithm, ranking using this latter algorithm was mainly in accordance with results obtained from the GeNorm algorithm. Indeed, the 15 genes previously identified as suitable RGs by GeNorm occupied the first 16 positions of the NormFinder ranking. Moreover, the 5 most stable genes (*rpoD*/*proC*, *fabD*, *gmk*, and *rho*) were the same according to these two algorithms and the delta-Ct method^35^ ranked in a different order. Lastly, in order to determine a global ranking, we used the RefFinder^36^ web-based tool to calculate the geometric mean of the four previous rankings. We found that *gmk* is the most stably expressed gene followed by *proC*, *fabD*, *rpoD*, *nadB*, *rho*, *thrA*, *ribD*, *mutL rpoB*, *adk* and *tmk*. Those twelve genes were considered stable enough to be employed as RGs by the four previously detailed methods and therefore were the best candidates for RT-qPCR experiment in *Y*. *pestis*. These selected genes were among those with the lowest dispersion C_q_ parameters.

**Table 2:** Stability values and ranking of 29 candidate reference genes.

Ranking	GeNorm		NormFinder		BestKeeper		Delta Ct		RefFinder	
	Gene	M value	Gene	Stab. value	Gene	Std dev	Gene	Av. STDEV	Gene	Score
1	***rpoD | proC***	0.313	***gmk***	0.213	*16S rRNA*	0.36	***gmk***	0.76	***gmk***	2.38
2			***fabD***	0.289	***nadB***	0.42	***fabD***	0.77	***proC***	3.08
3	***fabD***	0.451	***proC***	0.346	***rpoB***	0.44	***proC***	0.80	***fabD***	3.83
4	***gmk***	0.479	***rpoD***	0.411	***tmk***	0.51	***rho***	0.83	***rpoD***	3.85
5	***rho***	0.508	***rho***	0.419	***mutL***	0.51	***rpoD***	0.84	***nadB***	5.05
6	***thrA***	0.532	***nadB***	0.429	*cysG*	0.54	***nadB***	0.84	***rho***	6.32
7	***adk***	0.547	***thrA***	0.430	*tpiA*	0.62	***thrA***	0.85	***thrA***	8.01
8	***ribD***	0.560	***ribD***	0.448	***gmk***	0.64	***ribD***	0.86	***ribD***	8.24
9	***nadB***	0.572	***adk***	0.473	***ribD***	0.69	***adk***	0.86	***mutL***	8.41
10	***mutL***	0.583	***mutL***	0.519	***proC***	0.69	***mutL***	0.89	***rpoB***	9.32
11	***tmk***	0.611	*secA*	0.586	***rpoD***	0.75	*secA*	0.90	***adk***	9.60
12	***rpoB***	0.631	*ftsZ*	0.626	*ftsZ*	0.77	*gyrB*	0.93	***tmk***	9.78
13	*tpiA*	0.651	***tmk***	0.641	*hcaT*	0.81	*ftsZ*	0.96	*16S rRNA*	11.18
14	*ftsZ*	0.671	*gyrB*	0.642	***thrA***	0.82	***rpoB***	0.97	*tpiA*	12.16
15	*secA*	0.690	***rpoB***	0.644	***adk***	0.87	*tpiA*	0.97	*ftsZ*	12.72
16	*gyrB*	0.707	*tpiA*	0.649	***rho***	0.89	***tmk***	0.97	*secA*	14.14
17	*gapA*	0.725	*gapA*	0.677	*recA*	0.90	*gapA*	0.99	*cysG*	15.13
18	*glnA*	0.745	*gyrA*	0.772	***fabD***	0.91	*glnA*	1.02	*gyrB*	15.94
19	*gyrA*	0.762	*cysG*	0.775	*cysK*	0.94	*gyrA*	1.03	*gapA*	17.70
20	*rpsL*	0.782	*glnA*	0.785	*gapA*	0.96	*cysG*	1.05	*hcaT*	19.68
21	*tolB*	0.798	*hcaT*	0.799	*rpoS*	1.01	*tolB*	1.08	*gyrA*	20.08
22	*dnaK*	0.813	*recA*	0.807	*secA*	1.17	*hcaT*	1.08	*glnA*	20.45
23	*cysG*	0.829	*tolB*	0.817	*tolB*	1.19	*recA*	1.09	*recA*	21.32
24	*recA*	0.844	*16S rRNA*	0.857	*gyrB*	1.21	*rpsL*	1.09	*tolB*	21.98
25	*hcaT*	0.858	*rpsL*	0.871	*gyrA*	1.28	*16S rRNA*	1.11	*rpsL*	24.08
26	*16S rRNA*	0.874	*dnaK*	0.880	*dnaK*	1.29	*dnaK*	1.11	*dnaK*	24.94
27	*rpoA*	0.908	*rpoA*	1.295	*glnA*	1.33	*rpoA*	1.42	*cysK*	25.41
28	*cysK*	0.953	*cysK*	1.380	*rpsL*	1.38	*cysK*	1.55	*rpoS*	26.75
29	*rpoS*	1.017	*rpoS*	1.794	*rpoA*	1.78	*rpoS*	1.89	*rpoA*	27.49

Ranking are based on results from GeNorm, NormFinder, BestKeeper and Delta-Ct. M value: stability value calculated from GeNorm; Stab. Value: calculated from NormFinder; std dev: standard deviation calculated from BestKeeper, Av. STDEV: average standard deviation calculated from Delta-Ct method. Score calculate from RefFinder; In bold, the 12 genes validated in every conditions.

### Set of stable RGs for RT-qPCR

According to the MIQE guidelines^13^, the assessment of the stability of the RGs for each experimental condition should be performed prior to conduct the transcriptomic study. Furthermore, only one gene may not be sufficient unless its invariant expression has been proven. Vandesompele *et al*.^32^ proposed to test up to five candidate RGs and to determine how many are necessary for a correct normalization. Thus, we propose the panel of the five following genes *gmk*, *proC*, *fabD*, *rpoD* and *nadB*, as an universal panel for transcriptional studies in *Y*. *pestis*. However, even if these five genes had been validated in each condition of temperature and growth phase (data not shown) and could be proposed for all studies, they might not be the best one for a normalization process in specific conditions. Therefore, we performed the RefFinder analysis by grouping the data according to the temperature or the state of growth (Table S3). By picking the five best-ranked genes belonging to the 12 previously validated genes in each condition, an optimal panel can be proposed for each condition (Table 3). These five genes are ranked from the most stable to the less stable in our conditions but should be used together and reevaluated for each new experiment.

**Table 3:** Five recommended reference genes in function of the state of growth.

State of growth	All conditions	Exponential	Stationary	21 °C	26 °C	37 °C
Recommended reference genes	*gmk*	*fabD*	*nadB*	*rho*	*gmk*	*gmk*
	*proC*	*gmk*	*fabD*	*gmk*	*rho*	*proC*
	*fabD*	*nadB*	*gmk*	*nadB*	*fabD*	*nadB*
	*rpoD*	*proC*	*rpoD*	*mutL*	*nadB*	*mutL*
	*nadB*	*rpoD*	*proC*	*proC*	*proC*	*thrA*
**State of growth**	**Exponential 21** **°C**	**Exponential 26** **°C**	**Exponential 37** **°C**	**Stationary 21** **°C**	**Stationary 26** **°C**	**Stationary 37 °C**
Recommended reference genes	*gmk*	*gmk*	*proC*	*rho*	*rho*	*rho*
	*rho*	*fabD*	*gmk*	*fabD*	*rpoB*	*nadB*
	*fabD*	*rho*	*rpoD*	*gmk*	*gmk*	*fabD*
	*nadB*	*nadB*	*adk*	*nadB*	*nadB*	*gmk*
	*mutL*	*rpoB*	*fabD*	*mutL*	*thrA*	*rpoD*

The five recommended reference genes correspond to the five best-ranked genes belonging to the 12 previously validated genes in each condition. They are recommended in exponential or stationary phase regardless the temperature and for each temperature regardless the state of growth. Five genes are also proposed for each specific condition of temperature and state of growth.

### Comparison between normalized expression level for the genes of interest

In *Yersinia enterocolitica*, the expression of *acrAB* (YPO3132-33) gene encoding a RND efflux pump has been reported to be regulated by temperature and growth phase^37^. In *Y*. *pestis*, in addition to *acrAB*, five putative RND efflux pumps genes have been identified: YPO0420-21, YPO1000-01, YPO2847-48-49, YPO3043 and YPO3482-83. We assessed the influence of the temperature and the growth phase on their expression.

**Figure 3:**
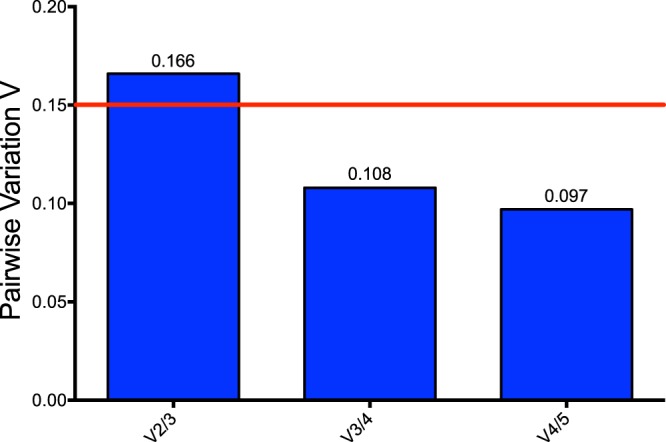
Pairwise variation calculated by GeNorm algorithm. The pairwise variation (V_n_/V_n+1_) was calculated between the normalization factors NF_n_ and NF_n+1_ to determine the optimal number of reference genes for accurate RT-qPCR normalization. The cut-off value was 0.15.

**Figure 4:**
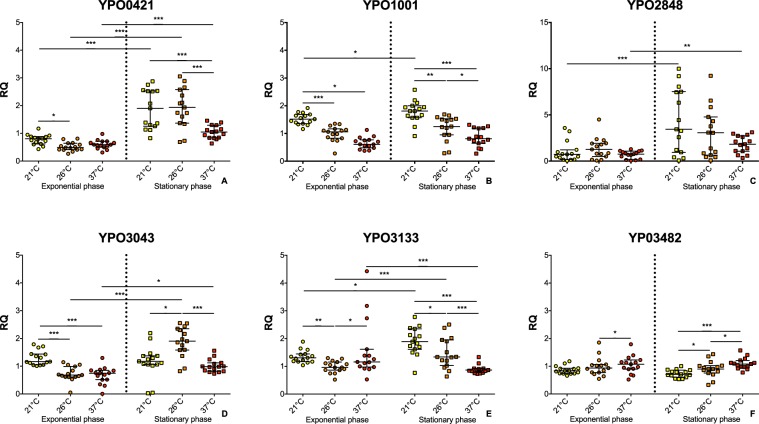
Expression of the gene of RND efflux pumps in function of temperature and state of growth. All data are presented as the relative expression levels obtained by normalization of the relative quantity for the genes of interest (A: YPO0421; B: YPO1001; C: YPO2848; D: YPO3043; E: YPO3133; F: YPO3482). Median and interquartile gap were shown. Statistical analysis was performed using Kruskal Wallis test followed by a Holm-Bonferroni procedure: *p < 0.05, **p < 0.01, ***p < 0.001.

Based on the expression of five ranked most stable genes *gmk*, *proC*, *fabD*, *rpoD* and *nadB* given by RefFinder according to the GeNorm method, the pairwise variation (V_n_/V_n+1_) was calculated between the normalization factors NF_n_ and NF_n+1_ to determine the optimal number of required RGs. The genes inclusion has been stopped when the pairwise variation reached the cut-off value below 0.15, in our case after the fourth ranked gene. Therefore, *gmk*, *proC*, *fabD* and *rpoD* have been chosen as RGs (Fig. 3). The normalized gene of interest’s Relative Quantity (RQ) position and dispersion data have been reported in Table S4 and represented in Fig. 4. Due to the non-normal distribution of the data the non-parametric Kruskal and Wallis test followed by a Holm-Bonferroni procedure (α risk set at 5%) have been used.

The results obtained showed that the regulation of *acrAB* in *Y*. *pestis* is function of temperature and state of growth and that RND efflux pumps genes expression is sensitive to the same stimuli. However, these trends that have been observed only from transcriptomic data do not allow us to extrapolate any phenotypic change or make any biological interpretation. For example, locus tag YPO1001 gene (Fig. 4B) transcription diminished with temperature with a 2.5-fold median decrease at the exponential phase and a 2.2-fold median decrease at the exponential phase between 21 °C and 37 °C. At the opposite, locus tag YPO3482 gene (Fig. 4F) showed a moderate increase in function of the temperature (only significant at the stationary phase). The other genes expression levels (Fig. 4A,C,D,E) showed less clear patterns. Despite the lack of formal hypothesis concerning the gene regulation, some statistically significant variations in RND efflux pumps expression have been demonstrated to depend on temperature and growth phase. Bearing in mind that the total efflux capacity of the bacterium is dependent of multiples pumps, these variations probably have an important role on the physiology of *Y*. *pestis* during the different steps of its life cycle. This role should be evaluated through further studies, where the same methodology could be applied.

### Effect of normalizing with non-validated RGs

To illustrate the impact of the choice of the most appropriate RGs, we performed two alternative normalizations of the gene expression data. On one hand, we used the expression of the *16S RNA* gene, the most commonly used normalizer^30^, and on the other hand we used the geometrical mean of the expression of our five last ranked genes, namely *rpsL*, *dnaK*, *cysK*, *rpoS* and *rpoA*. The results are presented respectively in Figs S3 and S4. By these two normalizations, the number of statistically significant results dramatically dropped from 32 to 14 and 16 respectively. Furthermore, three differences have been found as statistically significant with the *16S RNA* normalization, but not with our RGs.

## Discussion

The selection and validation of RGs are the most critical steps for transcriptomic studies^13^. For the highly pathogenic bacterium *Y*. *pestis*, a set of validated RGs might be proposed for exploring gene expression in many experimental conditions. However our results do not support the established practices and are in accordance with Bustin *et al*. who demonstrated that publications using RT-qPCR have deficiencies in the validation of experimental designs even in high impact factor journals^15^. Indeed, we observed that several genes such as *16S RNA*, *gyrA*, *recA* or *rpoA*, which are commonly used to normalize the expression of targeted genes^30^ are unsuitable for RT-qPCR in *Y*. *pestis*. We showed that growth conditions increased internal variability even in the most stable expressed genes and that the use of not-suitable RGs exposes the author to the risk of misinterpretation of results. It emphasizes the absolute necessity of choosing the right RGs to increase the signal-to-noise ratio and to validate results in the specific experimental condition. To our best knowledge, this is the first study validating RGs in *Y*. *pestis* on various growth conditions. More generally, we highlight that the previous validation of RGs before performing transcriptomic studies on prokaryotes is not standard enough while this step should be the prior requirement. This methodology is more conducted on eukaryotes, reason why all the algorithms we used were developed for humans, animals or plants.

The case of *16S RNA* deserves to be more scrutinized because it is the most used gene on RT-qPCR in bacteria^30^. Even if this gene was found as the most stable gene by the BestKeeper algorithm, it did not reach the minimum requirements to be accepted by the other algorithms. In our study, the normalization using *16S RNA* expression did not allow us to highlight as many differences as our validated standardization and even more identified differences that most probably do not exist. The suitability of *16S RNA* as a RG was already questioned^38^. Moreover, while the MIQE recommendations state that the abundances should show a strong correlation with the total amounts of mRNA present in the samples^13^, *16S RNA* has a very high level of transcription with about a 10^5^-fold ratio between its transcripts and the targeted genes mRNA. For these reasons, *16S RNA* cannot be recommended as a suitable RG at least under our conditions. This illustrates the necessity of performing validation tests for each experimental condition and using several algorithms to perform this test.

The need for suitable RGs does not limit to RT-qPCR experiments but it may be extend to the recent advances in molecular biology as reverse transcription digital PCR (RT-dPCR). Through “The Digital MIQE Guidelines”, Huggett *et al*., who signed the MIQE Guidelines in 2009, emphasized the necessity of justifying the number and the choice of RGs^39^ for RT-dPCR. Even if absolute quantification obtained by dPCR have been proven to be more precise and reproducible than qPCR, these two technologies have been recently qualified as complimentary to obtain the best results for any experiment^40^.

In conclusion, this methodology allowed us to observe slight variations of efflux pump gene’s expression in *Y*. *pestis*. These differences could not be highlighted in previous transcriptomic studies using microarray expression data in *Y*. *pestis*^41–43^. The risk of bias is limited by using a validated set of RGs to normalize RT-qPCR data and following the MIQE recommendations with rigorous quality controls. This experimental workflow will authorize us to explore not only the interplay between the different active efflux systems by measuring their expression under various conditions but more widely all the transcriptomic changes in *Y*. *pestis* during its complete cycle of life.

## Methods

All steps are designed to follow the MIQE guidelines^13^. Graphical representation has been made using Prism® 6 (GraphPad Software, Inc.).

### Bacterial strain and growth conditions

All experiments with live agents were realized in a Biosafety Level 3 laboratory. *Yersinia pestis* CO92 strain^44^ was cultivated on Trypticase soja plates (Biomérieux) at 26 °C for 24 hours. Bacteria were grown overnight at experimental temperatures (21 °C, 26 °C or 37 °C) in a Luria-Bertani (LB) Lennox broth (Becton-Dickinson), then diluted in a fresh LB Lennox broth at OD_600_ of 0.010 and grown for an additional time until the culture reaches an OD_600_ around 0.600 [0.582; 0.650] for the exponential phase and an OD_600_ around 2.00 [1.815; 2.198] for the stationary phase (Table S5). A spectrophotometer BioPhotometer® Plus (Eppendorf) was used for OD determination. The MIQE guidelines^13^ identified the sample acquisition as the first potential source of experimental variability in RT-qPCR experiment. In order to control sample-to-sample variation, we processed five independent biological replicates for each condition (30 in total).

### RNA isolation and cDNA preparation

At each defined growth’s state, RNA was stabilized with the RNAprotect® Cell Reagent (Qiagen). Bacteria were lysed and RNA was purified with the RNeasy® Lipid Tissue Mini Kit (Qiagen). Samples were also treated with the RNase-Free DNase Set (Qiagen) for 10 min in the column. Extraction products have been stored at −80 °C. RNA concentration [150.8 ng/µL; 375.1 ng/µL] and purity was determined in duplicate using a NanoDrop 2000 spectrophotometer (Thermo Scientific) with 1.5 µL of product. Both OD_260/280_ [2.13 and 2.20] and OD_260/230_ [1.80; 2.49] ratios around 2 were considered as “pure” according to the manufacturer’s recommendations. RNA integrity has been verified using a Bioanalyzer® 2100 (Agilent) with a RNA Nano Chip (Agilent) and the RNA integrity number [9.8; 10] showed nucleic acid quality close to the maximal value^45^ (Table S5). 1 µg RNA in a total reaction volume of 50 µL was used to prepare cDNA using the Reverse Transcription Core kit 500 RT-RTCK-05 (Eurogentec) with random nonameric primers. The initial step lasted 10 min at 25 °C and was followed by the RT step 30 min at 48 °C and the inactivation step 5 min at 95 °C. cDNA were stored at −20 °C. Because the RT step is known to be an important source of external variation^21^, three separated reverse transcriptions were performed for each extraction product (90 in total) and only one manufactured kit was used for all the procedure. A pool of cDNA was constituted by adding the same quantity of each RNA extract. This pool has been used as an internal calibrator for the qPCR experiment. A no-RT control with no reverse transcriptase has been performed to validate the absence of genomic DNA contamination.

### Selection of candidate RGs, genes of interest and primer design

For each identified gene, a pair of primers was designed using the Oligo7 primer analysis software (DBA Oligo Inc.)^46^ based on the published genome^44^ (Table 3). The genes of interest were those coding all potential RND efflux pumps in *Y*. *pestis*, based on a homology study of the AcrB protein^47,48^ using the COG database^49^. The specificity of the primers were verified *in silico* using BLAST (https://blast.ncbi.nlm.nih.gov/Blast.cgi) then *in vitro* as well as the lack of primer dimers by the presence of a single melting curve point after a qPCR, showing a single product for each pair of primers^50^.

### Quantitative PCR

qPCR was performed in a LightCycler® 480 (Roche) using the LightCycler® 480 SYBR Green I Master (Roche) in 96 wells white PCR microplate and a sealing film (Fisher scientific). The reaction mixture, which was 20 µL in total volume, contained 10 µL of LightCycler® 480 SYBR Green I Master 2 × (Roche), an equal quantity depending on the reaction of forward and reverse primer at 10 µmol/L and 1 µL of cDNA at the appropriate dilution. The mixture was filled to 20 µL by PCR grade water. The PCR program was started from one step of pre-incubation at 95 °C for 5 min, followed by 45 amplification cycles. Each cycle was started by a denaturation step at 95 °C for 10 s and followed by an annealing step at a specific temperature for a specific time depending on the reaction. The amplification step lasted 5 s at 72 °C. The last step was a melting curve analysis. For each run, 2 No Template Controls (NTC) have been performed. The quantity of primers and cDNA as well as the annealing time and temperature were determined to optimize the PCR efficiency (Table S6) as explained below.

### Determination of the optimal quantitative real-time PCR conditions and the reaction efficiency

We choose to express data obtained from the LightCycler® 480 Software release 1.5.1.62 (Roche) as C_q_ values^51^ calculated by the 2(-Delta Delta C(T)) method^52^. PCR conditions for each candidate reference gene were determined to optimize the PCR efficiency by the generation of a three-point standard curve based on a two-fold dilution series of the cDNA pool. The efficiency was calculated from the slope of the standard curve generated for each run in the following equation E = 10^(−1/slope)^, where E = 2 corresponds to 100% efficiency and E = 1 to 0%^22^. The efficiency for a specific dilution ratio was obtained from the standard curve passing by the two-fold upper and lower dilution. Because low PCR efficiency and high differences between PCR efficiencies can lead to dramatic quantification error, the E value should be as close as 2 as possible and homogenous for all the studied genes^53^. A value between 1.900 and 2.100 (90 and 110% respectively) is usually considered as suitable^26^. PCR parameters including the dilution of the cDNA sample were determined for each candidate reference gene to reach an efficiency of the reaction as close as possible from 2. All PCR parameters are reported in Table S6. The calculated efficiencies for the candidate RGs varied from 1.966 to 2.025 (96.6% to 102.5%) and from 1.957 to 2.007 (95.7% to 100.7%) for the genes of interest. The mean for the candidate reference genes was 1.986 with a standard deviation of 0.013 and respectively 1.989 and 0.026 for the genes of interest showing a high PCR efficiency level (close to 100%) and a good homogeneity between the different reactions. It is important to note that efficiency close to 2 is very important to obtain because some of algorithms as BestKeeper and Delta-Ct methods as well as the RefFinder web-tool use non-transformed data. Efficiency too different to 100% could induce some bias. The internal variability, estimated by calculating the difference of C_q_ values between two identical and representative samples represented by the pool previously described was always lower than 0.30.

### Evaluation and validation of candidate RGs stability

In order to avoid inter-run variability^51^, the samples were tested in one unique run in addition of four wells containing cDNA pool and two wells of negative control. No replicate has been performed at this step. The stability of reference gene candidates has been evaluated using four methods: Genorm^32^, NormFinder^34^, BestKeeper^33^ and the comparative Delta-Ct method^35^ combined in the online package RefFinder^36^.

- The GeNorm algorithm calculates for each candidate reference gene the pairwise variation with all other reference gene candidates. The control gene stability measurement M is defined as the average pairwise variation of one gene with all the other candidates. With the stepwise exclusion of the highest M value, the two most stable genes can be selected. Vandesompele *et al*. recommended to use at least the three most stable genes and to stepwise include the next most stable gene until the inclusion has no significant effect anymore^32^.
- The NormFinder algorithm combines the intra and inter-group variations to estimate the systematic error, which is induced by the use of one reference gene candidate. Andersen *et al*. assumed that the average of the candidate genes expression would show less systematic variation than the individual candidate genes and advised to use several genes for normalization^34^.
- The BestKeeper algorithm discards the less stable candidate RGs by calculating the standard deviation (SD) and a coefficient of variance of the derived crossing points. Pfaffl *et al*. advised to use the geometric mean of the best candidate RGs to normalize the expression data of the genes of interest. All those best genes are combined into an index. The Pearson correlation coefficient (r) and the probability p are calculated to describe the relation between the index and each candidate^33^.
- For Silver *et al*., the Delta-Ct algorithm is based on the comparison of the expression of all possible pairs of genes within each pair-wise sample. The stability of the candidate RGs is estimated from the range and the percentiles of this difference and used to rank the candidate RGs^35^.

For using GeNorm and NormFinder, expression data had to be preliminary transformed into relative quantities (RQ) by taking into account the previously described efficiency according to the equation RQ = E^∆Ct(control-sample)^. On the opposite side, BestKeeper and Delta-Ct methods used non-transformed data. On the RefFinder^36^ algorithm, Xie *et al*. used the C_q_ data of each sample for each gene and processed them with the four previous algorithms. The global comprehensive ranking of the candidate RGs is based on the geometric mean of the ranking from the four algorithms. Expression data are not transformed and efficiency is supposed to be very close to 2.

### Application to the study of efflux pumps

In order to study the expression of the genes of interest, we used the same methodology for the primer design and for the optimization of the qPCR than those developed for the candidate RGs. However, the RGs have been determined by using only the GeNorm algorithm with a cut-off value at 0.15. Indeed, this algorithm is the only one, which takes into account the PCR efficiency, indicates the number of references gene to use and provides a cut-off control value. The RQ of the genes of interest have been normalized using the geometrical mean of the RQ of the selected RGs. Statistical analysis has been performed by employing R® software (The R foundation).

## Supplementary information

Supplementary Information

## Data Availability

The data that support the findings of this study are available from the corresponding author upon reasonable request.
