# Supplementary material for: Transcriptomic studies and assessment of Yersinia pestis reference genes in various conditions
Source: Sci Rep. 2019 Feb 21;9:2501. doi: 10.1038/s41598-019-39072-x (PMC6385181; doi:10.1038/s41598-019-39072-x)
Supplement: Supplementary file 1 — Supplementary Information [file 41598_2019_39072_MOESM1_ESM.pdf]

Title

Transcriptomic studies and assessment of *Yersinia pestis* reference genes in various conditions

Authors

Lionel Koch[1,2,3], Thomas Poyot[1], Marine Schnetterle[1,3], Sophie Guillier[1,3], Estelle Soulé[1,3], Flora Nolent[1,3], Olivier Gorgé[1,3], Fabienne Neulat-Ripoll[1,3], Eric Valade[1,2,3], Florent Sebbane[4], Fabrice Biot[1,3]*

[1] Institut de Recherche Biomédicale des Armées (IRBA), Brétigny-sur-Orge, France

[2] Ecole du Val de Grace (EVDG), Paris, France

[3] Aix Marseille Univ, INSERM, SSA, IRBA, MCT, Marseille, France

[4] Inserm, Univ. of Lille, CNRS, CHU Lille, Institut Pasteur de Lille, U1019-UMR8204-CIIL-Center for Infection and Immunity of Lille, F-59000 Lille, France

*Corresponding author: Fabrice V. Biot, Institut de Recherche Biomédicale des Armées, Département de Biologie des Agents Transmissibles, Unité de Bactériologie, U1261-UMR_MD1, B.P. 73, 91220 Brétigny-sur-Orge, France, Phone (+33)1-78-65-10-60, Fax (+33)1-78-65-19-60, email: fbiot.irba@gmail.com

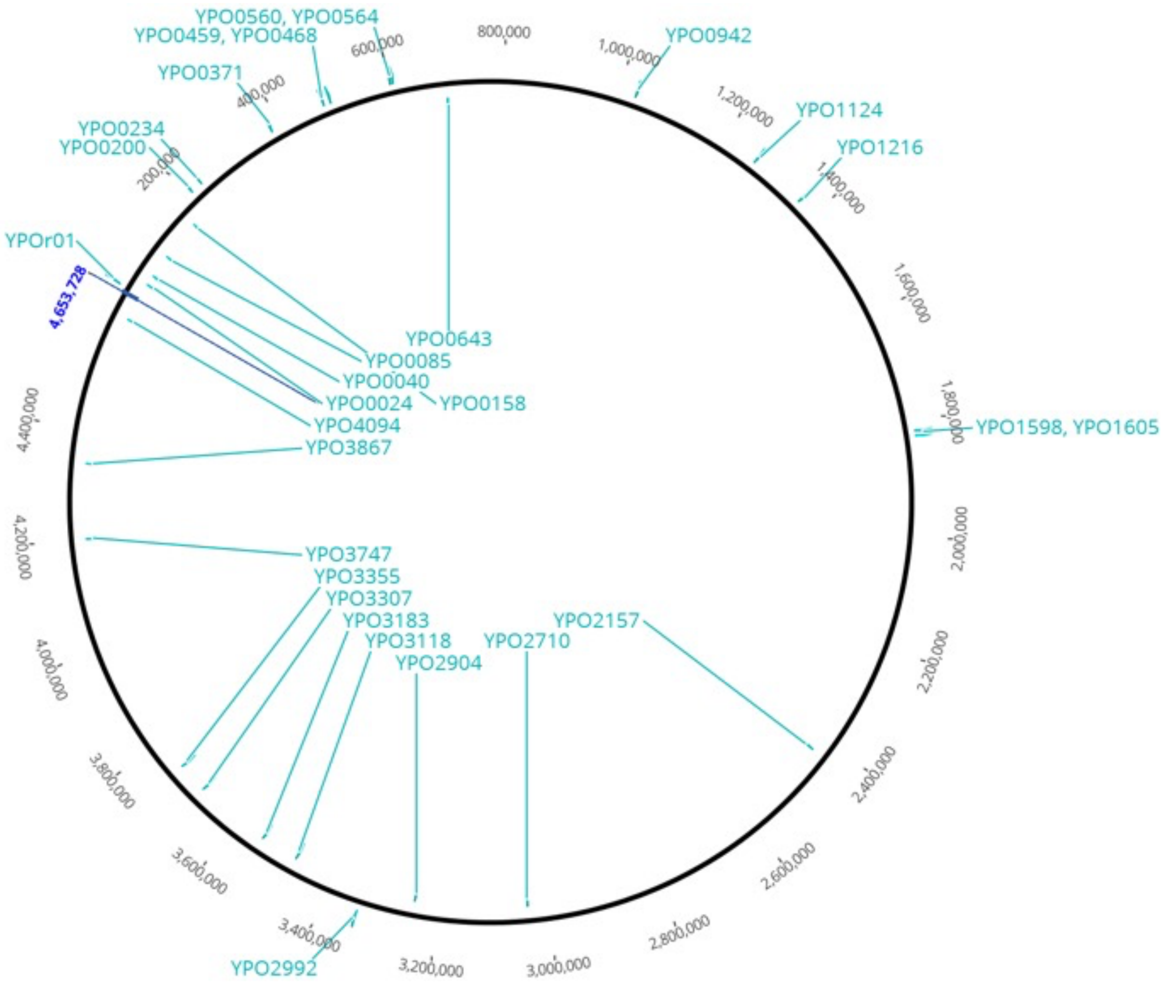

Figure S1: Loci of candidate reference genes on the *Yersinia pestis* chromosome.

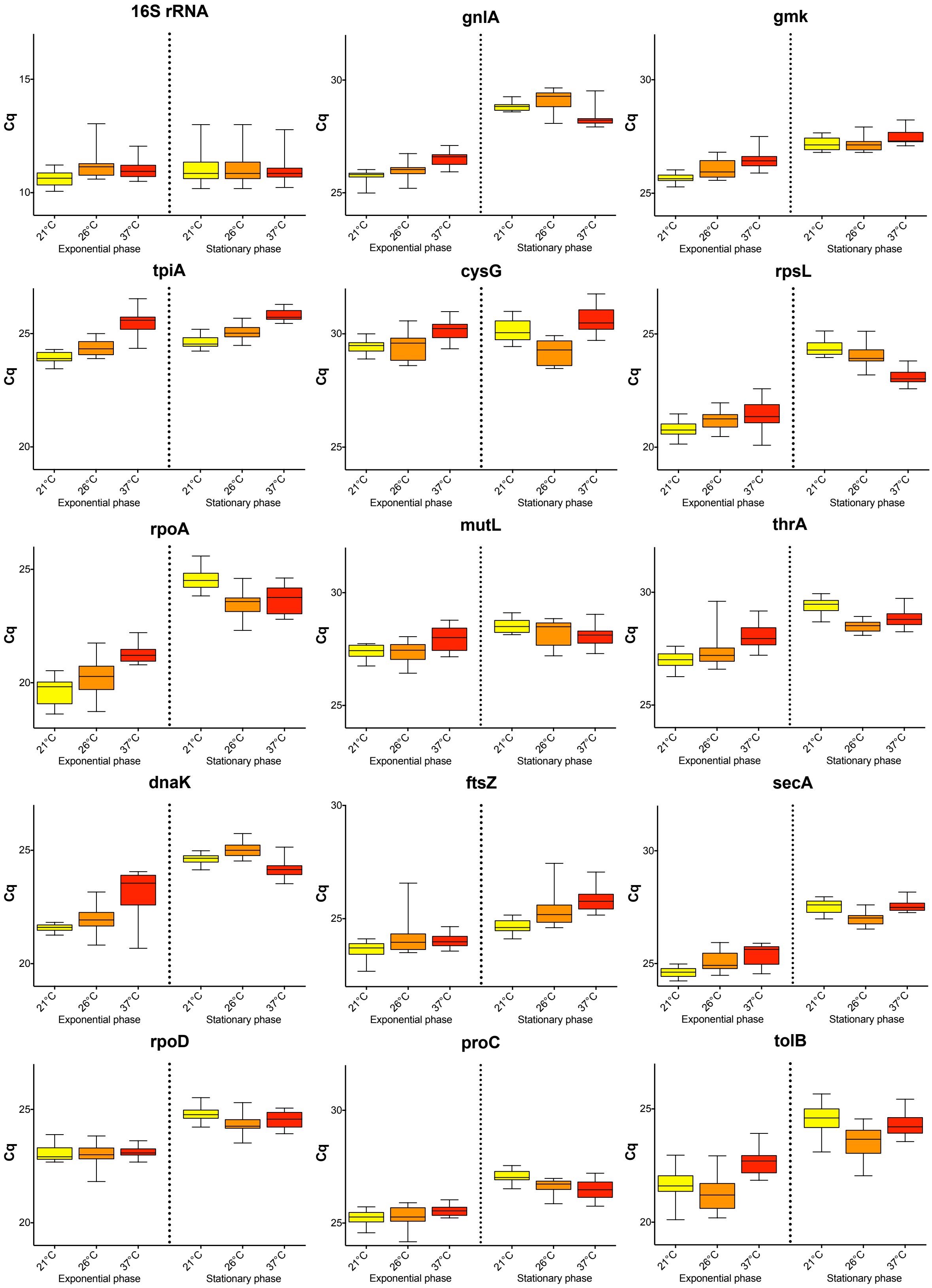
16S rRNA
Cq
15
10
21°C 26°C 37°C
Exponential phase
21°C 26°C 37°C
Stationary phase
gnlA
Cq
30
25
21°C 26°C 37°C
Exponential phase
21°C 26°C 37°C
Stationary phase
gmk
Cq
30
25
21°C 26°C 37°C
Exponential phase
21°C 26°C 37°C
Stationary phase
tpiA
Cq
25
20
21°C 26°C 37°C
Exponential phase
21°C 26°C 37°C
Stationary phase
cysG
Cq
30
25
21°C 26°C 37°C
Exponential phase
21°C 26°C 37°C
Stationary phase
rpsL
Cq
25
20
21°C 26°C 37°C
Exponential phase
21°C 26°C 37°C
Stationary phase
rpoA
Cq
25
20
21°C 26°C 37°C
Exponential phase
21°C 26°C 37°C
Stationary phase
mutL
Cq
30
25
21°C 26°C 37°C
Exponential phase
21°C 26°C 37°C
Stationary phase
thrA
Cq
30
25
21°C 26°C 37°C
Exponential phase
21°C 26°C 37°C
Stationary phase
dnaK
Cq
25
20
21°C 26°C 37°C
Exponential phase
21°C 26°C 37°C
Stationary phase
ftsZ
Cq
30
25
21°C 26°C 37°C
Exponential phase
21°C 26°C 37°C
Stationary phase
secA
Cq
30
25
21°C 26°C 37°C
Exponential phase
21°C 26°C 37°C
Stationary phase
rpoD
Cq
25
20
21°C 26°C 37°C
Exponential phase
21°C 26°C 37°C
Stationary phase
proC
Cq
30
25
21°C 26°C 37°C
Exponential phase
21°C 26°C 37°C
Stationary phase
tolB
Cq
25
20
21°C 26°C 37°C
Exponential phase
21°C 26°C 37°C
Stationary phase

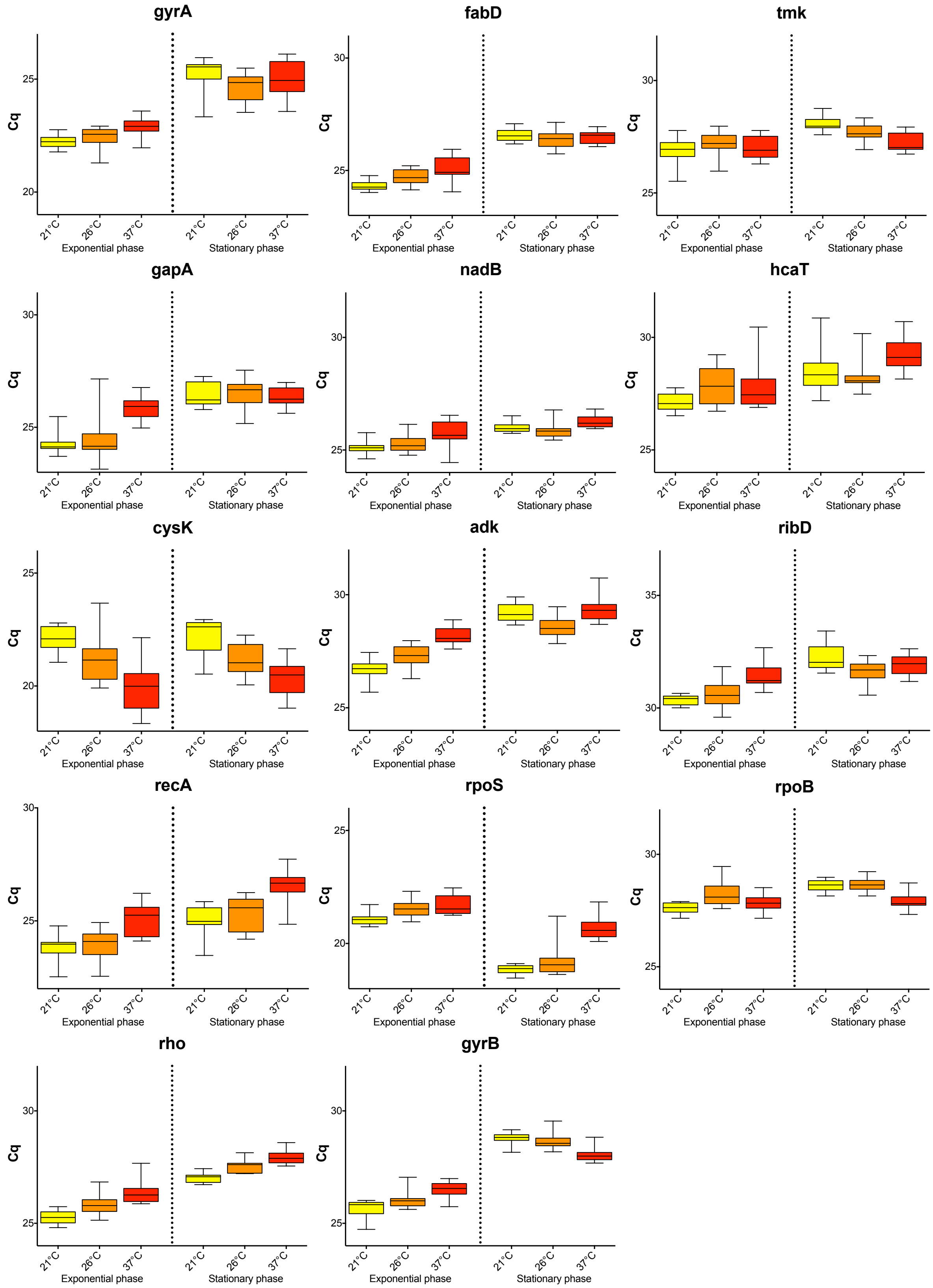

Figure S2: Expression level for the 29 candidate reference genes in all samples in function of the state of growth and temperature. Expression level was represented by the C_q values of each gene. Ebox indicated the 25th and 75th percentiles and the whiskers caps represented the maximum and minimum values. The line across the boxes indicated the median.

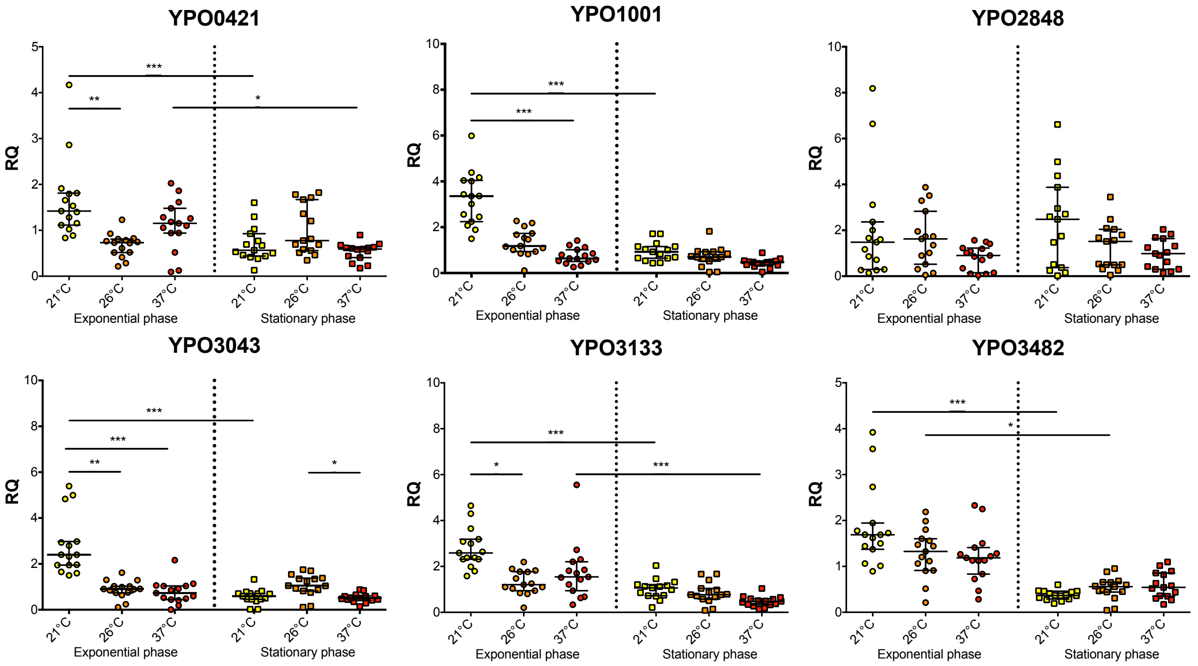

Figure S3: Expression of the gene of RND efflux pumps in function of temperature and state of growth with an alternative normalization using *16S RNA* gene expression. All data are presented as the relative expression levels obtained by normalization (*16S RNA* gene) of the relative quantity for the genes of interest (A: YPO0421; B: YPO1001; C: YPO2848; D: YPO3043; E: YPO3133; F: YPO3482). Median and interquartile gap were shown. Statistical analysis was performed using Kruskal Wallis test followed by a Holm-Bonferroni procedure: *p < 0.05, **p < 0.01, ***p < 0.001.

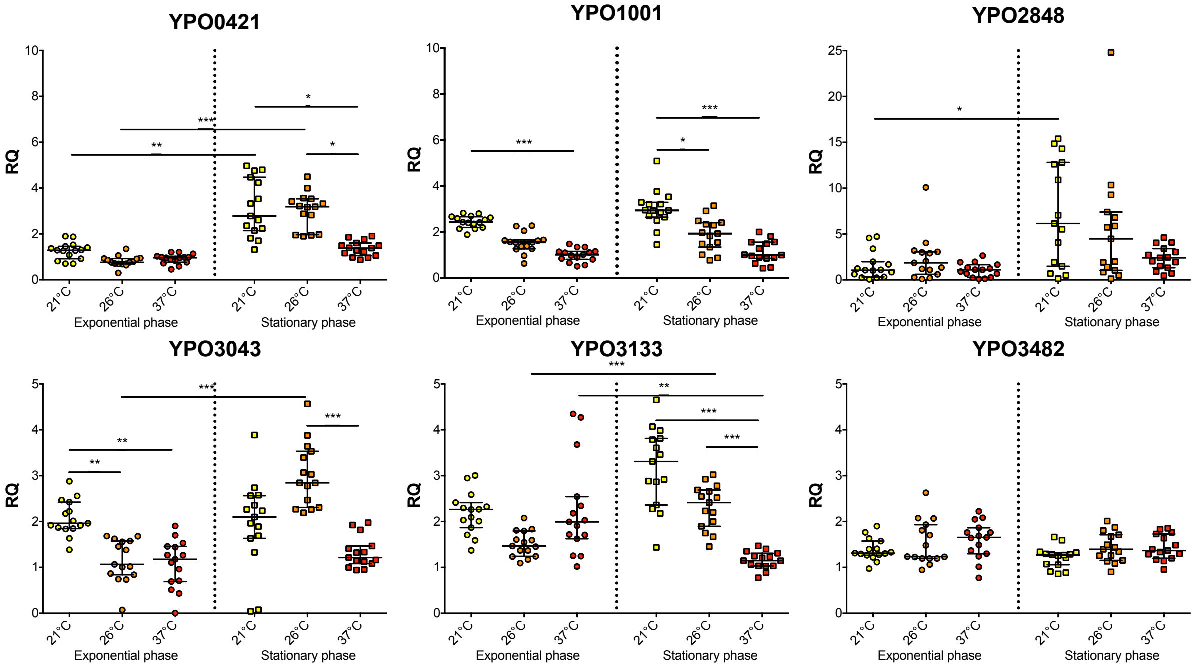

Figure S4: Expression of the gene of RND efflux pumps in function of temperature and state of growth with an alternative normalization using the geometrical mean of *rpsL*, *dnaK*, *cysK*, *rpoS* and *rpoA*. All data are presented as the relative expression levels obtained by normalization (*rpsL*, *dnaK*, *cysK*, *rpoS* and *rpoA*) of the relative quantity for the genes of interest (A: YPO0421; B: YPO1001; C: YPO2848; D: YPO3043; E: YPO3133; F: YPO3482). Median and interquartile gap were shown. Statistical analysis was performed using Kruskal Wallis test followed by a Holm-Bonferroni procedure: *p < 0.05, **p < 0.01, ***p < 0.001.

Gene	Min	1st Q	Med	3rd Q	Max	IG	Range
16S rRNA	10.06	10.65	10.83	11.07	13.04	0.42	2.98
glnA	24.99	26.03	27.51	28.73	29.65	2.70	4.66
gmk	25.28	25.98	26.87	27.27	28.23	1.29	2.95
tpiA	23.45	24.31	24.86	25.48	26.54	1.17	3.09
cysG	28.47	29.44	29.77	30.25	31.76	0.81	3.29
rpsL	20.09	21.09	22.58	23.92	25.13	2.83	5.04
rpoA	18.62	20.47	22.26	23.78	25.59	3.31	6.97
mutL	26.43	27.46	27.84	28.47	29.11	1.01	2.68
thrA	26.26	27.43	28.30	28.90	29.94	1.47	3.68
dnaK	20.68	21.8	23.90	24.65	25.74	2.85	5.06
ftsZ	22.68	23.89	24.48	25.18	27.44	1.29	4.76
secA	24.23	24.90	26.23	27.44	28.17	2.54	3.94
rpoD	21.82	23.00	23.80	24.57	25.52	1.57	3.70
proC	24.17	25.37	25.86	26.79	27.55	1.42	3.38
tolB	20.11	21.88	23.01	24.16	25.66	2.28	5.55
gyrA	21.29	22.51	23.50	25.00	26.11	2.49	4.82
fabD	24.02	24.64	25.77	26.48	27.14	1.84	3.12
tmk	25.52	26.95	27.27	27.89	28.76	0.94	3.24
gapA	23.14	24.49	25.93	26.61	27.54	2.12	4.40
nadB	24.44	25.26	25.80	26.04	26.82	0.78	2.38
hcaT	26.52	27.25	28.00	28.83	30.86	1.58	4.34
cysK	18.34	20.34	21.14	22.08	23.67	1.74	5.33
adk	25.69	27.47	28.26	28.98	30.74	1.51	5.05
ribD	29.59	30.58	31.52	31.98	33.42	1.40	3.83
recA	22.53	24.12	24.89	25.67	27.73	1.55	5.20
rpoS	18.47	19.11	20.95	21.41	22.46	2.30	3.99
rpoB	27.16	27.75	28.09	28.59	29.46	0.84	2.30
rho	24.81	25.80	26.83	27.57	28.59	1.77	3.78
gyrB	24.73	26.00	27.36	28.55	29.55	2.55	4.82

Table S1: C_q value median and dispersion parameters for each candidate reference gene. Min: minimum C_q value; 1st Q: first quartile value; Med: median value; 3rd Q: third quartile value; Max: maximum C_q value; IG: interquartile gap.

Gene	State	T°	Min	1st Q	Med	3rd Q	Max	IG	Range
16S rRNA	Exp	21°C	10.06	10.35	10.64	10.82	11.22	0.47	1.16
		26°C	10.60	10.79	11.14	11.23	13.04	0.44	2.44
		37°C	10.50	10.71	10.94	11.12	12.05	0.41	1.55
	Stat	21°C	10.45	10.63	10.69	10.88	11.31	0.25	0.86
		26°C	10.18	10.73	10.85	11.17	13.00	0.45	2.82
		37°C	10.23	10.70	10.85	11.06	12.78	0.36	2.55
glnA	Exp	21°C	24.99	25.71	25.81	25.87	26.03	0.16	1.04
		26°C	25.20	25.87	26.03	26.12	26.74	0.25	1.54
		37°C	25.93	26.35	26.60	26.69	27.10	0.34	1.17
	Stat	21°C	28.59	28.67	28.83	28.91	29.26	0.25	0.67
		26°C	28.08	28.92	29.28	29.43	29.65	0.52	1.57
		37°C	27.92	28.10	28.22	28.29	29.52	0.20	1.60
gmk	Exp	21°C	25.28	25.56	25.64	25.79	26.03	0.23	0.75
		26°C	25.57	25.77	25.94	26.44	26.81	0.67	1.24
		37°C	25.89	26.27	26.43	26.59	27.50	0.32	1.61
	Stat	21°C	26.80	26.97	27.13	27.39	27.66	0.42	0.86
		26°C	26.80	26.92	27.13	27.28	27.92	0.37	1.12
		37°C	27.09	27.28	27.30	27.65	28.23	0.36	1.14
tpiA	Exp	21°C	23.45	23.80	23.90	24.11	24.30	0.31	0.85
		26°C	23.90	24.09	24.33	24.62	25.00	0.53	1.10
		37°C	24.35	25.20	25.59	25.71	26.54	0.52	2.19
	Stat	21°C	24.23	24.45	24.54	24.79	25.19	0.34	0.96
		26°C	24.48	24.91	25.02	25.26	25.68	0.34	1.20
		37°C	25.45	25.64	25.72	25.97	26.29	0.34	0.84
cysG	Exp	21°C	28.89	29.27	29.48	29.61	30.00	0.34	1.11
		26°C	28.60	29.03	29.59	29.79	30.57	0.76	1.97
		37°C	29.34	29.91	30.23	30.35	30.98	0.44	1.64
	Stat	21°C	29.44	29.80	30.05	30.41	30.99	0.61	1.55
		26°C	28.47	28.78	29.29	29.68	29.92	0.90	1.45
		37°C	29.71	30.32	30.48	31.04	31.76	0.72	2.05
rpsL	Exp	21°C	20.14	20.60	20.76	21.03	21.47	0.43	1.33
		26°C	20.47	20.93	21.25	21.38	21.96	0.46	1.49
		37°C	20.09	21.11	21.35	21.72	22.58	0.61	2.49
	Stat	21°C	23.96	24.11	24.29	24.60	25.13	0.50	1.17
		26°C	23.19	23.83	23.92	24.30	25.12	0.48	1.93
		37°C	22.58	22.93	23.02	23.27	23.81	0.34	1.23

Gene	State	T°	Min	1st Q	Med	3rd Q	Max	IG	Range
rpoA	Exp	21°C	18.62	19.18	19.82	20.02	20.53	0.84	1.91
		26°C	18.73	19.73	20.28	20.71	21.75	0.98	3.02
		37°C	20.79	20.98	21.21	21.47	22.21	0.49	1.42
	Stat	21°C	23.83	24.25	24.51	24.83	25.59	0.57	1.76
		26°C	22.31	23.23	23.58	23.72	24.60	0.48	2.29
		37°C	22.80	23.28	23.76	24.10	24.62	0.82	1.82
mutL	Exp	21°C	26.75	27.22	27.43	27.63	27.74	0.41	0.99
		26°C	26.43	27.11	27.45	27.67	28.05	0.57	1.62
		37°C	27.16	27.49	28.01	28.29	28.78	0.81	1.62
	Stat	21°C	28.14	28.28	28.50	28.74	29.11	0.47	0.97
		26°C	27.20	27.75	28.49	28.66	28.85	0.91	1.65
		37°C	27.30	27.80	28.12	28.24	29.04	0.44	1.74
thrA	Exp	21°C	26.26	26.79	27.01	27.25	27.61	0.47	1.35
		26°C	26.59	26.95	27.20	27.51	29.60	0.56	3.01
		37°C	27.21	27.69	27.95	28.33	29.17	0.64	1.96
	Stat	21°C	28.69	29.26	29.47	29.64	29.94	0.38	1.25
		26°C	28.09	28.30	28.52	28.67	28.93	0.38	0.84
		37°C	28.25	28.60	28.80	29.03	29.73	0.43	1.48
dnaK	Exp	21°C	21.26	21.49	21.60	21.71	21.82	0.23	0.56
		26°C	20.82	21.66	21.93	22.19	23.16	0.53	2.34
		37°C	20.68	22.71	23.55	23.84	24.06	1.13	3.38
	Stat	21°C	24.14	24.48	24.65	24.76	24.98	0.28	0.84
		26°C	24.53	24.78	25.00	25.20	25.74	0.41	1.21
		37°C	23.53	23.95	24.15	24.32	25.14	0.37	1.61
ftsZ	Exp	21°C	22.68	23.47	23.71	23.90	24.11	0.43	1.43
		26°C	23.50	23.65	23.96	24.30	26.57	0.65	3.07
		37°C	23.57	23.84	23.98	24.20	24.65	0.36	1.08
	Stat	21°C	24.11	24.49	24.61	24.89	25.16	0.41	1.05
		26°C	24.60	24.89	25.18	25.52	27.44	0.64	2.84
		37°C	25.16	25.46	25.77	26.08	27.06	0.63	1.90
secA	Exp	21°C	24.23	24.46	24.62	24.78	24.98	0.32	0.75
		26°C	24.48	24.80	24.92	25.37	25.93	0.57	1.45
		37°C	24.55	25.00	25.63	25.75	25.90	0.76	1.35
	Stat	21°C	26.98	27.29	27.60	27.75	27.96	0.47	0.98
		26°C	26.53	26.80	27.02	27.13	27.60	0.33	1.07
		37°C	27.26	27.42	27.49	27.68	28.17	0.26	0.91

Gene	State	T°	Min	1st Q	Med	3rd Q	Max	IG	Range
rpoD	Exp	21°C	22.68	22.82	22.91	23.27	23.89	0.45	1.21
		26°C	21.82	22.85	23.00	23.23	23.83	0.38	2.01
		37°C	22.68	23.00	23.08	23.25	23.62	0.26	0.94
	Stat	21°C	24.22	24.63	24.77	24.96	25.52	0.33	1.30
		26°C	23.52	24.18	24.26	24.43	25.30	0.25	1.78
		37°C	23.93	24.28	24.57	24.78	25.06	0.49	1.13
proC	Exp	21°C	24.57	25.07	25.27	25.41	25.72	0.34	1.15
		26°C	24.17	25.11	25.27	25.68	25.90	0.57	1.73
		37°C	25.23	25.41	25.54	25.70	26.03	0.29	0.80
	Stat	21°C	26.52	26.93	27.02	27.25	27.55	0.31	1.03
		26°C	25.86	26.55	26.73	26.85	26.98	0.30	1.12
		37°C	25.75	26.16	26.48	26.74	27.21	0.57	1.46
tolB	Exp	21°C	20.11	21.41	21.60	22.00	22.96	0.59	2.85
		26°C	20.19	20.68	21.20	21.66	22.93	0.98	2.74
		37°C	21.85	22.26	22.70	22.93	23.92	0.67	2.07
	Stat	21°C	23.10	24.20	24.60	24.98	25.66	0.78	2.56
		26°C	22.05	23.16	23.66	24.00	24.56	0.84	2.51
		37°C	23.56	23.97	24.21	24.60	25.43	0.63	1.87
gyrA	Exp	21°C	21.78	22.02	22.23	22.38	22.76	0.36	0.98
		26°C	21.29	22.23	22.56	22.72	22.92	0.48	1.63
		37°C	21.96	22.72	22.91	23.05	23.59	0.33	1.63
	Stat	21°C	23.33	25.03	25.54	25.63	25.95	0.60	2.62
		26°C	23.53	24.42	24.85	25.03	25.49	0.62	1.96
		37°C	23.57	24.48	24.94	25.77	26.11	1.29	2.54
fabD	Exp	21°C	24.02	24.19	24.26	24.46	24.77	0.27	0.75
		26°C	24.14	24.51	24.68	24.97	25.21	0.46	1.07
		37°C	24.05	24.85	24.92	25.34	25.94	0.50	1.89
	Stat	21°C	26.18	26.39	26.54	26.78	27.08	0.38	0.90
		26°C	25.74	26.16	26.42	26.59	27.14	0.43	1.40
		37°C	26.06	26.33	26.57	26.68	26.95	0.35	0.89
tmk	Exp	21°C	25.52	26.71	26.94	27.15	27.78	0.44	2.26
		26°C	25.97	27.00	27.20	27.55	27.97	0.56	2.00
		37°C	26.29	26.61	26.90	27.38	27.78	0.77	1.49
	Stat	21°C	27.59	27.90	27.97	28.23	28.76	0.33	1.17
		26°C	26.93	27.50	27.63	27.97	28.34	0.47	1.41
		37°C	26.73	26.95	27.02	27.45	27.93	0.50	1.20

Gene	State	T°	Min	1st Q	Med	3rd Q	Max	IG	Range
gapA	Exp	21°C	23.71	24.08	24.13	24.29	25.48	0.21	1.77
		26°C	23.14	24.04	24.16	24.61	27.15	0.57	4.01
		37°C	24.97	25.52	25.93	26.16	26.77	0.65	1.80
	Stat	21°C	25.79	26.06	26.22	26.93	27.26	0.86	1.47
		26°C	25.17	26.27	26.67	26.87	27.54	0.60	2.37
		37°C	25.62	26.11	26.25	26.73	26.99	0.62	1.37
nadB	Exp	21°C	24.61	25.02	25.10	25.19	25.77	0.17	1.16
		26°C	24.77	25.08	25.19	25.50	26.14	0.42	1.37
		37°C	24.44	25.52	25.65	26.03	26.54	0.51	2.10
	Stat	21°C	25.74	25.84	25.95	26.08	26.52	0.24	0.78
		26°C	25.44	25.68	25.84	25.92	26.78	0.23	1.34
		37°C	25.95	26.03	26.19	26.39	26.82	0.35	0.87
hcaT	Exp	21°C	26.52	26.89	27.06	27.33	27.76	0.45	1.24
		26°C	26.72	27.28	27.83	28.53	29.23	1.26	2.51
		37°C	26.89	27.07	27.45	28.09	30.46	1.02	3.57
	Stat	21°C	27.19	27.90	28.34	28.80	30.86	0.90	3.67
		26°C	27.48	28.00	28.07	28.28	30.17	0.28	2.69
		37°C	28.15	28.81	29.11	29.72	30.70	0.91	2.55
cysK	Exp	21°C	21.05	21.72	22.09	22.58	22.79	0.86	1.74
		26°C	19.91	20.30	21.15	21.49	23.67	1.19	3.76
		37°C	18.34	19.11	19.99	20.52	22.14	1.42	3.80
	Stat	21°C	20.53	21.83	22.62	22.81	22.94	0.98	2.41
		26°C	20.05	20.69	21.03	21.83	22.25	1.15	2.20
		37°C	19.02	19.92	20.49	20.78	21.65	0.86	2.63
adk	Exp	21°C	25.69	26.58	26.73	26.92	27.45	0.34	1.76
		26°C	26.29	27.02	27.31	27.68	27.97	0.66	1.68
		37°C	27.60	27.92	28.07	28.35	28.89	0.43	1.29
	Stat	21°C	28.66	28.94	29.12	29.39	29.90	0.45	1.24
		26°C	27.84	28.26	28.51	28.86	29.47	0.60	1.63
		37°C	28.69	28.97	29.31	29.54	30.74	0.57	2.05
ribD	Exp	21°C	30.01	30.17	30.42	30.53	30.65	0.36	0.64
		26°C	29.59	30.32	30.56	30.86	31.84	0.54	2.25
		37°C	30.69	31.14	31.22	31.75	32.68	0.61	1.99
	Stat	21°C	31.55	31.82	32.03	32.45	33.42	0.63	1.87
		26°C	30.57	31.42	31.69	31.92	32.33	0.51	1.76
		37°C	31.18	31.65	31.97	32.17	32.63	0.52	1.45

Gene	State	T°	Min	1st Q	Med	3rd Q	Max	IG	Range
recA	Exp	21°C	22.53	23.72	23.97	24.04	24.78	0.32	2.25
		26°C	22.55	23.57	24.09	24.38	24.93	0.81	2.38
		37°C	24.11	24.64	25.25	25.60	26.22	0.96	2.11
	Stat	21°C	23.47	24.84	24.98	25.38	25.85	0.54	2.38
		26°C	24.19	24.58	25.58	25.93	26.25	1.35	2.06
		37°C	24.85	26.31	26.67	26.89	27.73	0.59	2.88
rpoS	Exp	21°C	20.74	20.91	21.06	21.18	21.72	0.27	0.98
		26°C	20.96	21.30	21.53	21.71	22.31	0.41	1.35
		37°C	21.25	21.38	21.53	22.00	22.46	0.61	1.21
	Stat	21°C	18.47	18.73	18.89	19.00	19.11	0.27	0.64
		26°C	18.63	18.80	19.06	19.34	21.21	0.54	2.58
		37°C	20.09	20.38	20.58	20.93	21.84	0.56	1.75
rpoB	Exp	21°C	27.16	27.47	27.63	27.80	27.90	0.33	0.74
		26°C	27.59	27.84	28.10	28.56	29.46	0.72	1.87
		37°C	27.16	27.63	27.83	28.01	28.52	0.38	1.36
	Stat	21°C	28.15	28.46	28.64	28.80	28.98	0.34	0.83
		26°C	28.15	28.46	28.64	28.79	29.23	0.34	1.08
		37°C	27.33	27.75	27.81	28.06	28.73	0.32	1.40
rho	Exp	21°C	24.81	25.09	25.26	25.48	25.74	0.39	0.93
		26°C	25.14	25.55	25.79	26.00	26.84	0.45	1.70
		37°C	25.87	26.01	26.26	26.50	27.67	0.50	1.80
	Stat	21°C	26.72	26.84	27.07	27.14	27.43	0.29	0.71
		26°C	27.21	27.25	27.61	27.66	28.14	0.41	0.93
		37°C	27.55	27.70	27.89	28.05	28.59	0.35	1.04
gyrB	Exp	21°C	24.73	25.56	25.83	25.91	26.02	0.35	1.29
		26°C	25.62	25.79	26.00	26.07	27.05	0.28	1.43
		37°C	25.74	26.31	26.55	26.69	26.99	0.38	1.25
	Stat	21°C	28.16	28.70	28.82	28.89	29.16	0.19	1.00
		26°C	28.18	28.47	28.56	28.78	29.55	0.31	1.37
		37°C	27.68	27.84	27.99	28.11	28.83	0.26	1.15

Table S2: C_q value median and dispersion parameters for each candidate reference gene in each growth condition. State: state of growth (Exp: exponential or Stat: stationary); T°: temperature of growth in °C Min: minimum C_q value; 1st Q: first quartile value; Med: median value; 3rd Q: third quartile value; Max: maximum C_q value; IG: interquartile gap.

	Total (all conditions)		Exponential (all temperatures)		Stationary (all temperatures)		21°C (all states of growth)		26°C (all states of growth)		37°C (all states of growth)		Exponential 21°C		Exponential 26°C		Exponential 37°C		Stationary 21°C		Stationary 26°C		Stationary 37°C	
1	***gmk***	2.38	***fabD***	2.45	***nadB***	1.41	***rho***	3.25	***gmk***	2.38	***gmk***	3.19	*secA*	2.21	*secA*	1.78	***proC***	1.32	*ftsZ*	2.45	***rho***	1.86	*secA*	1.57
2	***proC***	3.08	***gmk***	2.78	***fabD***	1.57	***gmk***	4.12	***rho***	3.62	***proC***	4.47	*tpiA*	2.34	***gmk***	3.15	*ftsZ*	2.11	***rho***	2.99	***rpoB***	2.00	***rho***	2.21
3	***fabD***	3.83	*rpoS*	3.00	***gmk***	2.28	*tpiA*	4.61	***fabD***	3.83	*cysG*	5.14	***gmk***	3.46	*tpiA*	3.74	*gyrB*	3.46	***fabD***	4.09	***gmk***	3.56	***nadB***	2.24
4	***rpoD***	3.85	***nadB***	3.76	*secA*	4.00	***nadB***	4.98	*secA*	4.23	***nadB***	5.47	***rho***	3.80	***fabD***	4.56	*rpoS*	3.72	*tpiA*	4.41	*secA*	3.87	*tpiA*	3.13
5	***nadB***	5.05	*gyrB*	5.83	***rpoD***	5.00	***mutL***	5.79	***nadB***	5.05	*tpiA*	5.53	***fabD***	3.98	*rpoS*	4.95	*rpoA*	5.89	***gmk***	4.46	*tpiA*	5.03	*gyrB*	4.16
6	*rho*	6.32	***proC***	5.97	***proC***	6.00	*ftsZ*	5.80	*tpiA*	5.32	***mutL***	6.64	***nadB***	5.83	*gyrB*	5.18	***gmk***	6.51	*rpoS*	4.95	***nadB***	5.24	***fabD***	6.48
7	*thrA*	8.01	*glnA*	6.67	*gyrB*	7.71	***proC***	6.16	***proC***	6.96	***thrA***	6.67	*glnA*	6.16	*glnA*	6.17	***rpoD***	6.61	*glnA*	6.70	*dnaK*	5.66	***gmk***	6.90
8	*ribD*	8.24	*rpsL*	8.43	*mutL*	8.92	*rpoB*	7.07	*rpoB*	7.34	*gyrB*	6.87	*dnaK*	7.60	***rho***	6.29	***adk***	7.42	***nadB***	6.88	***thrA***	5.76	*glnA*	8.49
9	*mutL*	8.41	***rpoD***	9.17	*thrA*	9.02	*fabD*	7.93	*adk*	7.36	*rpoB*	7.09	***mutL***	8.32	***nadB***	6.45	*glnA*	9.43	*gyrB*	7.95	*gyrB*	7.67	*rpsL*	8.92
10	*rpoB*	9.32	*gyrA*	10.02	*ribD*	10.16	*rpoD*	8.09	*rpoD*	9.17	*fabD*	8.11	*rpoB*	9.80	***rpoB***	8.46	***fabD***	9.60	***mutL***	8.09	*fabD*	9.46	***rpoD***	10.24
11	*adk*	9.60	*rho*	10.59	*rpoB*	10.89	*cysG*	9.08	*tmk*	9.53	*ribD*	8.96	*rpoS*	9.87	*rpsL*	9.21	*cysG*	10.34	*16S rRNA*	8.13	*rpsL*	11.80	*rpoB*	11.54
12	*tmk*	9.78	*16S rRNA*	10.91	*rho*	11.42	*ribD*	9.64	*ribD*	10.24	*tmk*	9.50	*cysG*	11.22	*rpoD*	11.58	*rpoB*	11.17	*secA*	10.10	*proC*	11.93	*mutL*	12.06
13	*16S rRNA*	11.18	*ftsZ*	11.13	*dnaK*	13.94	*tmk*	10.60	*mutL*	11.14	*adk*	9.64	*rpsL*	11.55	*proC*	12.49	*thrA*	12.49	*dnaK*	11.52	*tmk*	12.49	*gapA*	12.42
14	*tpiA*	12.16	*rpoB*	11.38	*adk*	14.14	*16S rRNA*	11.29	*thrA*	12.42	*rpoD*	9.64	*ftsZ*	14.06	*dnaK*	13.31	*mutL*	13.94	*proC*	12.10	*adk*	14.24	*proC*	14.89
15	*ftsZ*	12.72	*mutL*	12.15	*tmk*	14.83	*thrA*	12.06	*recA*	15.73	*rho*	12.27	*proC*	15.64	*gyrA*	13.73	*nadB*	14.16	*rpoB*	13.17	*glnA*	14.74	*thrA*	15.09
16	*secA*	14.14	*secA*	12.62	*glnA*	15.14	*adk*	14.34	*16S rRNA*	16.04	*gapA*	12.80	*ribD*	15.91	*adk*	15.47	*gyrA*	14.73	*rpsL*	14.97	*rpoD*	14.89	*ribD*	15.71
17	*cysG*	15.13	*cysG*	14.64	*gapA*	15.46	*gapA*	14.68	*gyrA*	16.85	*16S rRNA*	13.37	*gyrA*	16.71	*ribD*	17.96	*16s RNA*	15.55	*thrA*	15.98	*ribD*	17.24	*dnaK*	17.16
18	*gyrB*	15.94	*ribD*	16.30	*16S rRNA*	17.28	*recA*	17.23	*hcaT*	17.14	*glnA*	15.07	*16s RNA*	17.24	*mutL*	17.99	*tmk*	16.44	*rpoD*	17.96	*cysG*	17.74	*tmk*	17.46
19	*gapA*	17.70	*thrA*	17.73	*tpiA*	18.73	*secA*	17.79	*cysG*	17.49	*rpsL*	17.36	*thrA*	17.71	*tmk*	18.14	*tpiA*	17.94	*adk*	18.73	*gyrA*	19.49	*cysG*	17.72
20	*hcaT*	19.68	*tmk*	19.29	*rpsL*	19.95	*hcaT*	18.92	*ftsZ*	17.49	*ftsZ*	18.96	*gyrB*	18.33	*16s RNA*	18.26	*rho*	19.20	*rpoA*	20.73	*mutL*	20.48	*adk*	19.72
21	*gyrA*	20.08	*tpiA*	19.33	*gyrA*	21.23	*dnaK*	19.07	*gyrB*	18.70	*recA*	20.25	*hcaT*	20.99	*cysG*	20.97	*ribD*	20.19	*cysG*	20.75	*rpoA*	21.23	*rpoS*	20.86
22	*glnA*	20.45	*adk*	19.73	*rpoA*	22.21	*cysK*	19.27	*tolB*	19.93	*tolB*	21.25	*adk*	21.01	*ftsZ*	22.00	*rpsL*	22.19	*tmk*	20.92	*tolB*	21.40	*tolB*	21.71
23	*recA*	21.32	*rpoA*	23.72	*tolB*	22.23	*glnA*	20.08	*gapA*	21.49	*rpoS*	23.26	*rpoD*	21.99	*thrA*	22.48	*gapA*	22.45	*gapA*	23.48	*gapA*	23.21	*ftsZ*	22.49
24	*tolB*	21.98	*hcaT*	23.97	*cysG*	24.25	*gyrB*	21.62	*cysK*	21.84	*cysK*	23.59	*gapA*	23.31	*tolB*	24.00	*secA*	24.21	*gyrA*	24.48	*cysK*	24.22	*16s RNA*	23.64
25	*rpsL*	24.08	*recA*	23.99	*ftsZ*	24.48	*gyrA*	23.41	*rpsL*	22.14	*secA*	23.94	*tmk*	24.75	*rpoA*	25.25	*recA*	24.23	*ribD*	24.49	*hcaT*	24.47	*rpoA*	24.25
26	*dnaK*	24.94	*tolB*	25.75	*hcaT*	26.00	*tolB*	23.48	*dnaK*	24.43	*dnaK*	23.95	*rpoA*	25.20	*recA*	25.75	*tolB*	25.48	*recA*	25.97	*recA*	26.45	*recA*	26.00
27	*cysK*	25.41	*gapA*	27.25	*recA*	27.00	*rpoS*	25.74	*glnA*	25.93	*hcaT*	25.48	*cysK*	26.25	*hcaT*	27.25	*hcaT*	27.00	*tolB*	26.50	*16s RNA*	26.75	*cysK*	27.25
28	*rpoS*	26.75	*dnaK*	27.75	*rpoS*	28.00	*rpsL*	26.23	*rpoS*	27.66	*rpoA*	25.91	*recA*	28.22	*cysK*	28.25	*cysK*	28.00	*cysK*	28.00	*rpoS*	26.97	*gyrA*	28.25
29	*rpoA*	27.49	*cysK*	29.00	*cysK*	29.00	*rpoA*	28.25	*rpoA*	28.25	*gyrA*	25.97	*tolB*	28.25	*gapA*	28.49	*dnaK*	29.00	*hcaT*	29.00	*ftsZ*	29.00	*hcaT*	28.49

Table S3: Stability values and ranking of 29 candidate reference genes based on the results from the RefFinder web-based tool in all samples tested (Total) and for all the groups. Each RefFinder stability value represents the geometric mean of the ranking with the GeNorm, NormFinder, BestKeeper and Delta-Ct algorithms. In bold, the 5 recommended reference genes for each growth condition, which were the best ranked genes among the 12 previously validated.

Gene	State	Temp	Min	1st Q	Med	3rd Q	Max	IG	Range
YPO0421	Exp	21°C	0.43	0.65	0.81	0.88	1.17	0.23	0.74
		26°C	0.26	0.41	0.49	0.62	0.81	0.20	0.55
		37°C	0.31	0.53	0.59	0.68	0.98	0.16	0.67
	Stat	21°C	0.83	1.28	1.90	2.53	2.88	1.25	2.05
		26°C	0.69	1.49	1.93	2.47	3.06	0.98	2.37
		37°C	0.64	0.84	1.05	1.22	1.46	0.38	0.82
YPO1001	Exp	21°C	1.16	1.37	1.52	1.66	1.92	0.29	0.76
		26°C	0.28	0.85	1.06	1.15	1.35	0.31	1.07
		37°C	0.38	0.52	0.61	0.76	1.13	0.24	0.75
	Stat	21°C	0.91	1.60	1.81	1.97	2.58	0.37	1.67
		26°C	0.29	0.95	1.25	1.52	1.69	0.57	1.40
		37°C	0.27	0.67	0.81	1.19	1.31	0.52	1.04
YPO2848	Exp	21°C	0.04	0.28	0.73	1.10	3.58	0.82	3.54
		26°C	0.06	0.64	1.26	1.91	4.50	1.28	4.44
		37°C	0.06	0.34	0.76	1.02	1.29	0.68	1.23
	Stat	21°C	0.07	1.08	3.44	7.48	9.99	6.40	9.92
		26°C	0.08	0.74	3.07	4.55	9.22	3.81	9.14
		37°C	0.35	1.08	1.83	2.67	3.11	1.58	2.76
YPO3043	Exp	21°C	1.01	1.08	1.17	1.43	1.79	0.35	0.78
		26°C	0.05	0.61	0.68	0.91	1.15	0.30	1.09
		37°C	0.00	0.52	0.75	0.84	1.30	0.33	1.29
	Stat	21°C	0.03	1.06	1.17	1.37	2.19	0.31	2.16
		26°C	0.82	1.59	1.90	2.33	2.55	0.74	1.73
		37°C	0.73	0.83	0.98	1.10	1.56	0.27	0.83
YPO3133	Exp	21°C	1.04	1.18	1.31	1.44	1.89	0.26	0.85
		26°C	0.52	0.87	0.96	1.13	1.30	0.27	0.77
		37°C	0.53	0.99	1.16	1.50	4.43	0.51	3.90
	Stat	21°C	0.77	1.66	1.89	2.24	2.78	0.58	2.01
		26°C	0.64	1.12	1.34	1.93	2.51	0.81	1.87
		37°C	0.73	0.80	0.87	0.91	1.33	0.11	0.60
YPO3482	Exp	21°C	0.66	0.74	0.84	0.92	1.18	0.18	0.52
		26°C	0.55	0.78	0.94	1.05	1.86	0.27	1.31
		37°C	0.52	0.90	1.07	1.19	1.79	0.29	1.27
	Stat	21°C	0.54	0.64	0.71	0.83	1.00	0.20	0.47
		26°C	0.33	0.78	0.90	1.02	1.44	0.23	1.11
		37°C	0.77	0.98	1.08	1.17	1.57	0.19	0.80

Table S4: RQ (Relative Quantity) median and dispersion parameters for each gene of interest in function of the state of growth and temperature. State: state of growth (Exp: exponential or Stat: stationary); Temp: temperature of growth in °C Min: minimum C_q value; 1st Q: first quartile value; Med: median value; 3rd Q: third quartile value; Max: maximum C_q value; IG: interquartile

N°	T°	Phase	Ti	OD_{600}	C°	$OD_{260/280}$	$OD_{260/230}$	RIN
1	21	E	5	0.582	339.7	2.14	2.48	10
2	21	E	5	0.601	375.1	2.15	2.38	10
3	21	E	5	0.591	302.6	2.15	2.25	10
4	21	E	5	0.622	357.2	2.14	2.38	10
5	21	E	5	0.588	351.5	2.16	2.35	10
6	21	S	20	1.930	340.9	2.16	2.35	10
7	21	S	20	1.949	305.4	2.17	2.39	10
8	21	S	20	1.924	297.6	2.16	2.39	10
9	21	S	20	1.929	325.4	2.16	2.17	10
10	21	S	20	1.940	325.1	2.17	2.35	10
11	26	E	4	0.645	249.0	2.17	2.39	10
12	26	E	4	0.594	219.2	2.20	2.34	10
13	26	E	4	0.647	253.7	2.18	2.37	10
14	26	E	4	0.631	222.4	2.17	1.90	10
15	26	E	4	0.607	216.0	2.17	2.41	10
16	26	S	18	2.198	297.9	2.16	1.95	10
17	26	S	18	2.196	278.3	2.15	2.36	10
18	26	S	18	2.181	316.7	2.16	1.99	10
19	26	S	18	2.172	305.0	2.17	1.80	10
20	26	S	18	2.192	308.0	2.13	2.25	10
21	37	E	6	0.632	209.8	2.16	2.41	10
22	37	E	6	0.601	156.0	2.13	2.18	10
23	37	E	6	0.650	179.2	2.15	2.13	9.9
24	37	E	6	0.624	150.8	2.14	2.38	10
25	37	E	6	0.580	174.3	2.14	1.84	10
26	37	S	22	1.862	222.5	2.18	2.46	10
27	37	S	22	1.880	188.5	2.18	1.96	10
28	37	S	22	1.871	195.8	2.17	2.49	10
29	37	S	22	1.865	170.0	2.14	1.96	10
30	37	S	22	1.815	153.7	2.21	2.38	9.8

Table S5: Sample quality control: bacterial growth and RNA quantity, quality, purity and integrity. N°: unique sample number; T°: temperature of growth in °C; Phase E: exponential, S: stationary; Ti: time of growth in hours; OD_{600} is measured before extraction, RNA concentration (C°) in ng/µL and purity are measured immediately after extraction; the RNA integrity number (RIN) is measured on RNA conserved at -80°C.

Candidate reference gene													
Locus tag	RT product dilution	Primers C°	Addition of $MgCl_2$	Ann T°	Ann Ti	Length of product	Melting Temp	Slope of calibration curve	Error	Yintercept	PCR efficiency E	$\Delta(C_q)$	C_q of the NTC
YPOr01	2	0.5	0	54	5	77	83.8	-3.345	0.0214	13.33	1.990	0.02	39.15
YPO0024	8	0.5	0	60	5	83	78.3	-3.386	0.0121	27.77	1.970	0.14	39.35
YPO0040	8	0.625	0	50	5	77	82.8	-3.386	0.0121	27.77	1.973	0.03	39.35
YPO0085	8	0.5	0	58	5	147	84.3	-3.357	0.0140	25.06	1.986	0.06	>40
YPO0158	8	0.5	0	57	5	132	84.3	-3.367	0.0335	30.59	1.980	0.02	36.02
YPO0200	8	0.625	0	57	5	95	81.9	-3.320	0.0277	22.99	2.003	0.01	33.02
YPO0234	4	0.5	0	49	5	80	78.7	-3.392	0.0322	22.81	1.970	0.00	>40
YPO0371	8	0.5	0	51	5	87	78.8	-3.403	0.00157	28.17	1.966	0.05	>40
YPO0459	2	0.5	0	61	5	89	82.6	-3.395	0.00101	31.78	1.973	0.01	>45
YPO0468	8	0.5	0	54	5	82	81.6	-3.366	0.0163	23.64	1.980	0.06	>45
YPO0560	2	0.5	0	50	5	119	85.8	-3.331	0.0217	26.93	1.993	0.10	>40
YPO0564	8	0.5	0	58	5	87	81.2	-3.366	0.0157	27.59	1.983	0.05	>40
YPO0643	4	0.5	0	57	5	96	80.3	-3.379	0.00458	24.65	1.976	0.03	>40
YPO0942	4	0.5	0	61	5	70	79.5	-3.335	0.0204	27.45	1.976	0.01	>40
YPO1124	4	0.625	0	48	5	82	78.6	-3.345	0.0276	25.79	1.990	0.02	38.38
YPO1216	4	0.625	0	54	5	95	82.0	-3.342	0.0187	25.18	1.990	0.06	31.55
YPO1598	8	0.625	0	57	5	97	81.1	-3.327	0.0137	25.68	2.000	0.05	>45
YPO1605	8	0.625	0	58	5	93	80.5	-3.339	0.00874	28.20	1.993	0.04	>45
YPO2157	4	0.625	0	45	5	95	81.8	-3.323	0.00937	26.55	2.000	0.27	>45
YPO2710	8	0.5	0	57	5	108	82.7	-3.366	0.0260	26.03	1.983	0.19	>45
YPO2904	2	0.625	0	47	5	74	75.9	-3.311	0.0252	29.98	2.007	0.06	35.58
YPO2992	4	0.5	0	55	5	157	83.7	-3.303	0.00423	21.44	2.007	0.07	>45
YPO3118	8	0.5	0	54	5	101	85.7	-3.412	0.000149	25.72	1.997	0.20	39.16
YPO3183	8	0.5	0	52	5	143	84.7	-3.337	0.0226	30.52	1.976	0.24	>45
YPO3307	8	0.5	0	54	5	89	79.1	-3.297	0.0179	25.54	2.011	0.00	>40
YPO3355	2	0.5	0	57	5	109	86.1	-3.356	0.0112	22.56	1.983	0.02	38.72
YPO3747	8	0.625	0	58	6	116	81.5	-3.402	0.0146	28.62	1.970	0.04	>45
YPO3867	8	0.5	0	55	5	93	80.0	-3.367	0.00837	26.80	1.980	0.03	39.57
YPO4094	8	0.5	0	63	5	99	81.9	-3.357	0.0130	27.23	1.986	0.05	>45

Gene of interest													
Locus tag	RT product dilution[a]	Primers C°[b]	Addition of $MgCl_2$[c]	Ann T°[d]	Ann Ti[e]	Length of product[f]	Melting T°[g]	Slope of calibration curve[h]	Error[i]	Yintercept[j]	PCR efficiency E[k]	$\Delta(C_q)$[l]	C_q of the NTC[l]
YPO0421	4	0.625	0	52	6	137	83.0	-3.403	0.0200	31.00	1.966	0.10	>45
YPO1001	2	0.625	1	57	5	104	86.3	-3.342	0.0208	33.02	1.956	0.08	>45
YPO2848	8	0.625	0	58	5	116	85.9	-3.264	0.0286	31.51	2.025	0.27	39.19
YPO3043	4	0.5	0	55	5	97	80.1	-3.332	0.0424	32.89	1.997	0.00	>45
YPO3133	8	0.5	0	59	5	119	80.7	-3.371	0.00644	25.55	1.980	0.10	>45
YPO3482	4	0.5	0	60	5	112	84.0	-3.309	0.0335	32.99	2.007	0.09	>45

Table S6: PCR parameters and efficiency for the candidate reference genes and for the genes of interest. RT product dilution: dilution of the RT product for each gene; Primer C°: primer concentration in ng/µL; Addition of MgCl2 in the PCR mix in mM; Ann t°: temperature of annealing in °C; Ann Ti: time of annealing in second; Melting temperature in °C; Error and Yintercepts derived from these calibration curves were calculated by the LightCycler software; Efficiency were calculated with the formula $E = 10^{(-1/slope)}$; Δ(Cq): difference of C_q value between two identical representative samples (pool); C_q value of the NTC is the mean of the two NTC (NTC>40: C_q value between 40 and 45, NTC>45: no detection).
